# Synthesis of Nitrogen‐Doped Mesoporous Structures from Metal–Organic Frameworks and Their Utilization Enabling High Performances in Hybrid Sodium‐Ion Energy Storages

**DOI:** 10.1002/advs.201902986

**Published:** 2020-01-27

**Authors:** Gyu Heon Lee, Jeung Ku Kang

**Affiliations:** ^1^ Department of Materials Science and Engineering Korea Advanced Institute of Science and Technology (KAIST) 291 Daehak‐ro, Yuseong‐gu Daejeon 34141 Republic of Korea; ^2^ Graduated School of Energy, Environment, Water, and Sustainability (EEWS) NanoCentury KAIST Institute Korea Advanced Institute of Science and Technology (KAIST) 291 Daehak‐ro, Yuseong‐gu Daejeon 34141 Republic of Korea

**Keywords:** high energy density, hybrid sodium‐ion energy storage, N‐doped mesoporous structures, robust cycle stability, ultrafast charging

## Abstract

Sodium‐ion energy storage is of the most attractive candidate for commercialization adoption due to the safety and cost demands of large‐scale energy storage systems, but its low energy density, slow charging capability, and poor cycle stability are yet to be overcome. Here, a strategy is reported to realize high‐performance sodium‐ion energy storage using battery‐type anode and capacitor‐type cathode materials. First, nitrogen‐doped mesoporous titanium dioxide (NMTiO_2_) structures are synthesized via the controlled pyrolysis of metal–organic frameworks. They exhibit interconnected open mesopores allowing fast ion transport and robust cycle stability with nearly 100% coulombic efficiency, along with rich redox‐reactive sites allowing high capacity even at a high rate of ≈90 C. Moreover, assembling the NMTiO_2_ anode with the nitrogen‐doped graphene (NG) cathode in an asymmetric full cell shows a high energy density exceeding its counterpart symmetric cell by more than threefold as well as robust cycle stability over 10 000 cycles. Additionally, it gives a high‐power density close to 26 000 W kg^−1^ outperforming that of a conventional sodium‐ion battery by several hundred fold, so that full cells can be charged within a few tens of seconds by the flexible photovoltaic charging and universal serial bus charging modules.

## Graphical abstract

Hybrid sodium‐ion energy storage is reported with high energy density exceeding that of a symmetric supercapacitor by more than threefold, robust cycle stability over 10 000 cycles, and high power density close to 26 000 W kg^−1^ outperforming that of a sodium‐ion battery so that full cells can be charged within a few tens of seconds.

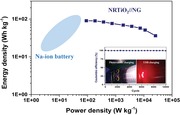

Beyond the increasing demand for electric vehicles (EVs) and portable devices,^1^ a rechargeable electrochemical energy storage system (ESS) is an essential component of many applications. Nowadays, the dominating ESS remains on a lithium‐ion battery (LIB),^2^ but the more challenging requirement for a future ESS continues to drive development of electrode materials with high energy density allowing prolonged operation upon a single charge, fast charging capability on excellent power density, and robust cycle stability over long charge–discharge cycles.^3^ As a solution to overcome the limitations by the low power density and short cycle stability of a LIB, a hybrid energy storage,^4^, ^5^, ^6^ which is referred to as an asymmetric electrochemical energy storage full cell assembled with battery‐type negative and capacitor‐type positive electrodes, is of great advantages as the next‐generation ESS. In hybrid energy storages, charges are asymmetrically stored by ion adsorption and pseudocapacitive interaction in cathode and anode materials, respectively, so that their charge and discharge processes can be controlled using different potential windows to increase energy density.^7^ Moreover, low‐cost hybrid energy storages can be developed using an earth‐abundant electrolyte including sodium (Na) ions.^8^, ^9^, ^10^, ^11^ However, many electrode materials suffer from several obstacles such as safety and poor capacity in a sodium‐ion electrolyte. Titanium dioxide (TiO_2_) is considered as a promising anode material for a hybrid energy storage as it is capable of being operated at a low potential that is advantageous for safety. The problem is that it gives poor electronic conductivity and ionic diffusivity, thus resulting in fast capacity fading and short cycle life.^12^, ^13^, ^14^, ^15^, ^16^, ^17^, ^18^, ^19^ Consequently, a new strategy that can overcome the current limitations of such an electrode material is expected to represent a great breakthrough in the development of high‐performance sodium‐ion electrochemical energy storages.

Herein, we realize hybrid sodium‐based electrochemical energy storages on a new paradigm strategy, where both a nitrogen‐doped mesoporous TiO_2_ (NMTiO_2_) structure synthesized through the controlled pyrolysis of a metal–organic framework (MOF) and also a nitrogen‐doped graphene (NG) were utilized as anode and cathode materials, respectively. The nitrogen‐doped mesoporous NMTiO_2_ structures are shown to give excellent energy density, high power density allowing ultrafast charging, and robust cycle stability over 10 000 cycles. An MOF has the secondary building units (SBUs) that could play potentially as active sites for electrochemical redox reactions. Also, its tunable porosity and functionality could offer fast ion transport pathways by enabling the facile accessibility of ion carriers to active sites. Moreover, the unique structures and properties of the NMTiO_2_ can be summarized as follows: 1) it contains open mesopores (≈4 nm) enabling an easy penetration of electrochemical Na‐ion carriers between the electrolyte and active sites; 2) ultrafine nanocrystals with nitrogen‐doped TiO_2_ units can be encapsulated inside its interior parts, which establish excellent stability at the fast charging rate such as ≈90 C (30 000 mA g^−1^) with negligible capacity reduction over a long cycle life; and 3) the heterogeneous N atoms in its matrix lead to enhanced electrochemical ion sorption/desorption during repeated charge–discharge cycles. Also, a NG, having many heterogeneous N atoms in the carbon matrix and facilitating the accessibility of anions, was employed as the cathode material to achieve high capacity. Furthermore, the NMTiO_2_//NG full cell is configured into a hybrid asymmetric configuration to demonstrate high energy density and robust cycle stability. Additionally, the flexible photovoltaic charging and USB charging modules are realized to demonstrate the ultrafast charging capability of NMTiO_2_//NG full‐cell devices.

A microwave‐assisted solvothermal method was used to prepare the NH_2_‐MIL‐125 (Ti) represented by the chemical formula of Ti_8_O_8_(OH)_4_(C_6_H_3_C_2_O_4_NH_2_)_6_ with a short reaction duration. During the synthesis, the functional groups of the NH_2_^−^ ions and Ti^4+^ ions in ligands (C_6_H_3_C_2_O_4_NH_2_)_6_ and SBUs (Ti_8_O_8_(OH)_4_) serve as the heterogeneous dopants and metal resources, respectively. In addition, the nanocages in MOFs play an important role in introducing mesopores.^20^, ^21^ The simple synthetic procedures of the anode and cathode materials for the full‐cell configuration and their energy storage mechanisms are shown in **Figure** **1** as schematics. The 3D mesoporous architecture having uniform sized nanoparticles (NPs) of nitrogen‐doped TiO_2_ units was successfully synthesized through the simple pyrolysis of MOFs in air condition, and Figure 1a shows that it maintains the original morphology of an MOF. The carbon species in the functional ligands were also removed during the thermal oxidation procedure, while mesopores were introduced in the remaining spaces. The amine functionalized–titanium MOFs were used as the template to obtain nitrogen‐doped mesoporous TiO_2_ NPs. It is found that the diffusion of sodium ions in a salt‐containing electrolyte is facilitated by the introduced mesopores in the NMTiO_2_. Furthermore, the nitrogen‐doped graphene was synthesized as a cathode material by plasma treatment with nitrogen gas (Figure 1b). The NG has a high active surface area from its 2D morphology. Also, the radius of 0.74 Å for a N atom is similar to that (0.77 Å) for a C atom so that N atoms are easily doped into the carbon matrix of graphene. Moreover, the electronegativity of 3.04 for N is higher than that of 2.5 for C so that heterogeneous nitrogen dopants modify the electronic distribution of the carbon matrix of graphene to improve electrical conductivity as well as provide abundant active sites for redox reactions.^22^, ^23^ The illustration of the NMTiO_2_//NG full cell and the corresponding energy storage mechanisms between the electrolyte and electrode atoms is presented in the schematics (Figure 1c,d). The titanium‐based MOFs were synthesized through the microwave‐assisted solvothermal technique having great advantages such as the high heating rate and short reaction time. In brief of methods, titanium isopropoxide (0.6 mmol) and 2‐aminoterephthalic acid (1.2 mmol) were dissolved in a mixed solvent of *N*,*N*‐dimethylformarmide (DMF) and methanol (MeOH) (20 mL, DMF:MeOH = 1:1 in volume), and then put into a 35 mL glass tube, which is capped by a rubber septum and placed in a microwave synthesizer. Next, the mixture was heated to 150 °C, held for 1 h, and then cooled to room temperature. The yellow product was washed with DMF and methanol several times. Also, the powder was dried and evacuated in vacuum oven at 60 °C. After then, the powder of the NH_2_‐MIL‐125 (Ti) was placed in an alumina boat under the steady air‐flow condition. Moreover, the sample was annealed to 350 °C, kept for 2 h, and then cooled to room temperature. The product is a light‐yellow‐colored powder, which is ascribed to nitrogen dopants playing to result in the decreased bandgap from 3.24 to 2.78 eV.^24^ The bandgap and coloring of the nitrogen‐doped TiO_2_ were investigated by UV–vis absorption spectra and real photo images, and Figure S1 (Supporting Information) shows the shift of observation spectra owing to the reduced bandgap by ≈0.54 eV and the light yellow color on nitrogen doping. It is notable that the nitrogen‐doped TiO_2_ NPs are formed, and the mesopores between particles are introduced during the thermal oxidation process. Mesopores in the negative electrode (Figure 1c) allow a facile access of Na^+^ ions to nitrogen‐doped TiO_2_ units, while heterogeneous N atoms in the carbon matrix of the NG facilitate the accessibility of ClO^4−^ ions in the positive electrode (Figure 1d).

**Figure 1:**
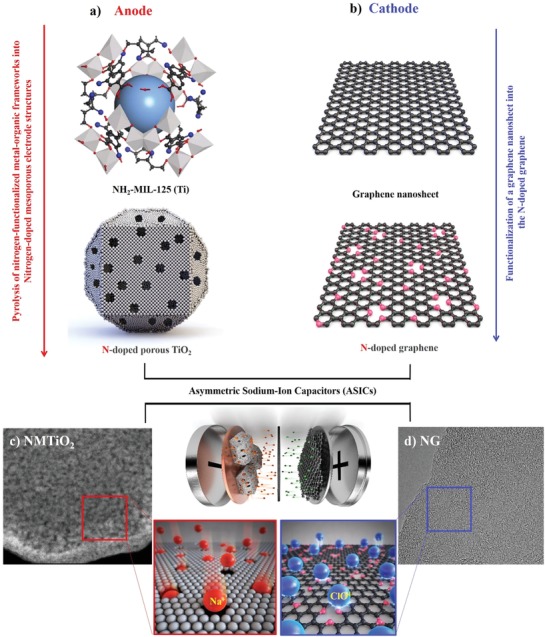
The schematics of all synthetic processes, mechanisms, and cell configurations. a) The schematics for the synthesis of a nitrogen‐rich mesoporous metal oxide anode material (NMTiO_2_) from the nitrogen‐functionalized metal–organic framework (NH_2_‐MIL‐125 (Ti)) via a facile pyrolysis process. The titanium ions were thermally oxidized with oxygen ions in the air so that it could maintain its original morphology. b) The schematics for synthesis of a nitrogen‐doped graphene (NG) cathode material via a nitrogen plasma treatment. The synthesized c) NMTiO_2_ anode and d) NG cathode materials assembled into a hybrid sodium‐ion energy storage full cell and the corresponding energy storage mechanisms for charge carriers in anode and cathode structures.

**Figure** **2**a shows the scanning electron microscope (SEM) image of the NH_2_‐MIL‐125 (Ti). The rectangular parallelepiped shapes with round edges are clearly observed. The mesoporous NMTiO_2_, which was synthesized at a low temperature through the pyrolysis of the NH_2_‐MIL‐125 (Ti), has a uniform shape and size, and its morphology is quite similar to that for the NH_2_‐MIL‐125 (Ti) (Figure S2, Supporting Information). The transmission electron microscope (TEM) and scanning TEM (STEM) images (Figure 2b; Figure S3, Supporting Information) further reveal a highly porous feature arising from the voids between its nanocrystals, implying that nanocrystals are homogeneously formed during the annealing procedure without leading to the structural collapse of the framework. The size of the NMTiO_2_ is also determined to be at an average size of 500 nm, and it has the average thickness of 140 nm. Figure 2c also shows a high‐resolution TEM (HRTEM) image elucidating the ≈7 nm size of a titanium nanocrystal with the lattice spacing of 0.346 nm corresponding to the (101) plane of the anatase phase. Additionally, the selected area electron diffraction (SAED) pattern of an individual framework (inset of Figure 2c) reveals the circular patterns showing a polycrystalline anatase.^25^ Such a highly crystalline framework ensures good mechanical robustness during electrode cycling. The constituents of the NMTiO_2_ are mainly Ti and O species, and also include nitrogen and carbon atoms, as confirmed from the STEM–energy dispersive spectroscopy (EDS) and X‐ray photoelectron spectroscopy (XPS) analyses (Figures S4 and S5, Supporting Information). The STEM–EDS mapping images further show that each constituent is uniformly distributed throughout the sample. The XPS spectra of the NMTiO_2_ sample show the distinct peaks corresponding to C 1s, O 1s, Ti 2p, and N 1s at ≈283, 529, 463, and 398 eV, respectively. Furthermore, the STEM–EDS analysis of the NG (Figure S6, Supporting Information) presents the uniformly distributed C, O, and N species as the constituent elements of the NG, while the XPS analysis (Figure S7, Supporting Information) of the NG for C 1s (≈284 eV), O 1s (≈531 eV), and N 1s (≈399 eV) demonstrates the sharp peak of N 1s indicating the existence of rich nitrogen atoms in the graphene sheets, so that they provide rich active sites for the electrochemical reactions with the ions in the electrolyte. In addition, the high‐resolution STEM image (Figure 2d) shows the open mesopores created at the TiO_2_ nanocrystal interfaces. Moreover, the high‐resolution STEM pore image in Figure 2e demonstrates that the mesopores with an average size of ≈4 nm were created. This feature is especially advantageous to give high rate capability and long‐term cycling stability due to the facilitated diffusion of electrolyte ions through the open mesopores. Furthermore, the crystallographic information for NMTiO_2_ and bare NH_2_‐MIL‐125 (Ti) structures was collected via the X‐ray diffraction (XRD). The high‐crystalline NH_2_‐MIL‐125 (Ti) was also confirmed from the sharp diffraction peaks attributed to the micropores in Figure S8a (Supporting Information).^26^ The diffraction peaks (Figure 2f) at 25.3°, 37.8°, 48.2°, and 54.6°, corresponding to (101), (004), (200), and (211) planes, are well matched with those for the anatase TiO_2_ (JCPDS card No. 21–1272). However, for the lower‐temperature‐annealed sample, the diffraction peaks of the rutile TiO_2_ (JCPDS card No. 21–1276) were not observed and the peaks are on more broad shapes and lower intensities, implying that the NMTiO_2_ could be synthesized to have a smaller particle size and a single anatase phase in the lower temperature. Also, a nitrogen sorption analysis at 77 K (Figure 2g) clarifies that the NMTiO_2_ possesses rich mesopores centered at the pore diameter of ≈4 nm, which is in a suitable size to enable the large uptake of electrolyte ions for fast ionic transport and also agree well with the pore sizes by the high‐resolution STEM images. Moreover, the isotherm analysis (Figure S8b, Supporting Information) shows that NH_2_‐MIL‐125 (Ti) has a high surface area (1146.3 m^2^ g^−1^). The nitrogen dopants in the NMTiO_2_ were further investigated by XPS analyses and the Fourier transform infrared spectroscopy (FT‐IR). The N 1s peak at 399.2 eV (Figure 2h) is attributed to N—Ti—N bonds formed by the substitutional doping of the O atoms in O—Ti—O bonds by N atoms. Furthermore, the Ti—N—O bonding formed the interstitial nitrogen dopants in the anatase TiO_2_ lattice is also confirmed by the peak at ≈401 eV.^27^, ^28^ Figure 2i and Figure S9 (Supporting Information) also show the band at ≈1080, 1250, 1390, and 1475 cm^−1^ attributed to the vibrations of Ti–N bonds as well as the band of 1630 cm^−1^ corresponding to the N–H bending vibration. The band of vibration from the surface‐absorbed NO_3_^−1^ was also confirmed at around 1390 cm^−1^. In addition, the broadband at ≈3400 cm^−1^ is shown to match with the spectra of the surface hydroxyl groups and absorbed water molecules. The band at ≈2340 cm^−1^ is attributed to the C=N=O asymmetric stretch.^29^, ^30^, ^31^ From these results, the appearance of the vibration bands related to the nitrogen bindings such as Ti—N, Ti—N—O, and N—Ti—N bonds supports that the nitrogen species were successfully incorporated in the lattice of TiO_2_ NPs.

**Figure 2:**
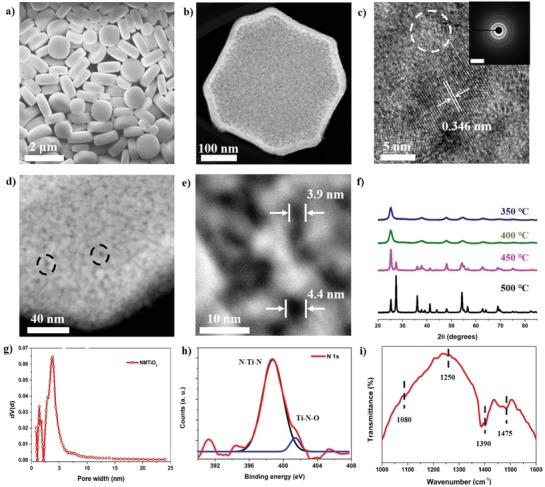
The analyses of the morphology, crystal structure, porosity, and chemical information for the NMTiO_2_ synthesized at a low temperature. a) The low‐resolution SEM image of the synthesized NH_2_‐MIL‐125(Ti). b) The STEM image at a low magnification and c) the lattice parameter of an anatase TiO_2_ (dotted circle: mesopore, inset: diffraction pattern). d) The high‐resolution STEM image of the NMTiO_2_ (dotted circles: mesopores). e) The STEM images of the NMTiO_2_ at 350 °C, where mesopores of ≈4 nm sizes are clearly observed. f) The XRD analysis of the NMTiO_2_ at various synthetic temperatures. g) The pore size distribution analysis of the NMTiO_2._ h) The XPS spectra of the N 1s peak and i) the FTIR spectra confirming the chemical bonding information of nitrogen species in the lattice of the NMTiO_2_.

To investigate the morphological influence on the cell performance, we synthesized the products at different processing temperatures and reaction times such as from 350 to 500 °C and from 30 min to 2 h, respectively. Figure S10 (Supporting Information) shows the thermal oxidation behavior of NH_2_‐MIL‐125 (Ti) under the air flow conditions. In the first range below ≈335 °C that is the crystallization temperature of titanium ions, we find that small amounts of surface‐absorbed water molecules and impurities were removed. After then, the crystallization of titanium ions and the combustion of carbon species were observed to be occurring up to ≈550 °C. These results imply that the thermal oxidation of titanium ions could be proceeded above 335 °C. The electrochemical performances for NMTiO_2_ and NG were also evaluated in coin‐type half‐cells with the sodium foils as both counter and reference electrodes. The STEM images of NMTIO_2_ samples (**Figure** **3**a) show that their morphologies are dependent on annealing temperatures. The sizes of the TiO_2_ nanoparticles at a high temperature are bigger than those at a lower temperature, and the mesopores in the interparticle interfaces are determined to be disappeared. However, the sample annealed at the lower temperature showed high‐porosity and small‐sized nanoparticles. The N_2_ adsorption/desorption analysis (Figure 3b) clarifies further that the NMTiO_2_ annealed at 350 °C leads to the highest surface area (170.3 m^2^ g^−1^) and a typical hysteresis isotherm feature (type IV).^32^ We also find from Figure S11 (Supporting Information) that the pore size distribution is dependent on an annealing temperature. The pore size distribution analysis shows that the samples annealed at low temperatures have smaller pore sizes, signaling that TiO_2_ frameworks annealed at low temperatures include more active sites for the electrochemical reactions. We find that the pores with an average size of ≈4 nm were significantly increased under the pyrolysis condition at the lowest temperature (350 °C). In addition, the electrochemical reactions of Na ions with the negative materials were characterized through the cyclic voltammetry (CV) measurements (Figure 3c) at a scan rate of 0.1 mV s^−1^ over the working potential window of 1–3 V versus Na/Na^+^. The CV curves present the cathodic and anode peaks at 1.75 and 2.1 V, corresponding to Na‐ion insertion and desertion reactions with the transitions between Ti^4+^ and Ti^3+^ chemical states.^33^ The rate capability and charge–discharge profile of the NMTiO_2_ electrode are determined to depend on a synthesis temperature (Figure 3d,e). The maximum reversible capacity of 269.7 mAh g^−1^ at ≈0.2 C is achieved and the capacity of 100.8 mAh g^−1^ is delivered even at a high rate of ≈90 C (30 000 mA g^−1^). The CV curves and rate performances support that the samples annealed at 350 °C for 30 min show the highest reversible capacity, thus, better than those at a higher temperature for a longer time. This indicates that the Na‐ion interaction into NMTIO_2_ nanocrystals should be mediated by open mesoporous channels introduced within ultrafine nanocrystals. In this reason, the sample annealed at 350 °C during the 30 min was selected as an anode material for a hybrid full‐cell device. In the charging and discharging processes, the discharging capacity is observed to be slightly higher than the charging capacity because the sodium insertion occurs during the discharging process increasing the voltage. The electrochemical impedance spectroscopy (EIS) analysis (Figure 3f) was also carried out to determine the nitrogen‐doping effect. It shows that the nitrogen‐doped mesoporous TiO_2_ structure has lower Ohmic resistance and charge transfer resistance values in the Nyquist plot than the bare anatase TiO_2_ sample. This indicates that the pristine TiO_2_ electrodes with poor electrical conductivities can be enhanced on nitrogen doping, so that the sodiation process should be facilitated by improved electrical conductivity as well as fast ionic accessibility with 3D interconnected mesopores in the TiO_2_ anode, thereby allowing high energy storage capacity and fast sodiation/desodiation.^34^, ^35^ Figure 3g shows further that the NMTiO_2_ electrode gives high capacity even at an ultrafast charging rate (≈90 C). The galvanostatic intermittent titration technique (GITT) measurements were also analyzed to determine the diffusion kinetics of Na^+^. Figure S12 (Supporting Information) shows the time–voltage profile of NMTiO_2_ during charging/discharging and the voltage plateaus for phase transition by sodium‐ion insertion were clearly observed during charging and discharging reactions. The difference of discharging behaviors was also shown in Figure S12b,c (Supporting Information). The discharging time of the 350 °C sample exhibits to be much longer than that of the 500 °C sample. Moreover, the 350 °C sample was found to accommodate the higher concentration of Na^+^ ions up to 1 mole fraction. From the time–voltage profiles of titrations in the voltage plateau region during discharging (Figure S12d,e, Supporting Information), we also observed that the 350 °C sample shows the much smaller Δ*E_τ_* of the transient voltage change during a titration current flux after the relaxation period. Consequently, these support that the nitrogen‐doped mesoporous TiO_2_ synthesized at the low annealing temperature can give the facilitated Na‐ion diffusion between the surface and the electrolyte.^36^, ^37^

**Figure 3:**
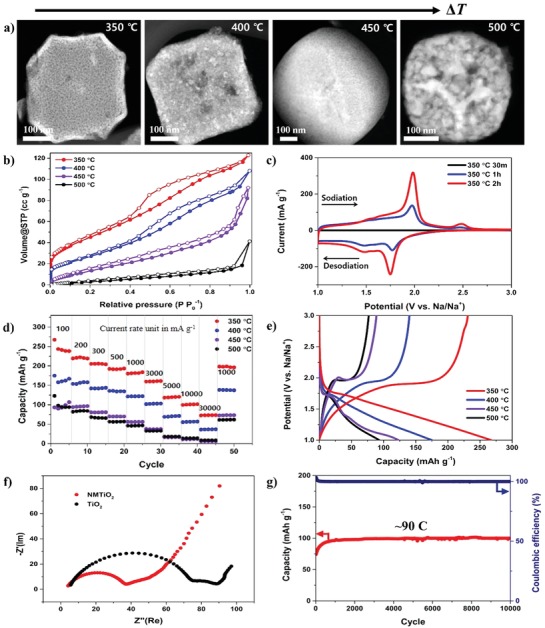
The electrochemical performances of half‐cells dependent on the porosities. a) The STEM images of NMTiO_2_ samples prepared at different temperatures. b) The N_2_ adsorption/desorption isotherms. c) The CV profiles of NMTiO_2_ samples on a synthetic time condition at 350 °C. d) The specific capacitances at different current densities (mA g^−1^). e) The charge–discharge profiles of NMTiO_2_ samples at different temperatures. f) The EIS analysis data of NMTiO_2_ and bare TiO_2_ samples. g) The cycling performance of the NMTiO_2_ at the current density of ≈90 C (30 000 mA g^−1^).

Figure S13 (Supporting Information) also clarifies the electrochemical performance of the NG cathode in a half‐cell configuration investigated with the Na foil as a counter electrode in the working potential window of 3–4.5 V. The CV curve shows a typical capacitive behavior and the gravimetric measurements demonstrate the maximum capacity of ≈78 mAh g^−1^.

We also realized the NMTiO_2_//NG sodium‐ion full‐cell device (**Figure** **4**a). The charge carriers such as Na^+^ and ClO^4−^ ions in the electrolyte can be easily diffused through the introduced mesopores in the NMTiO_2_ and electrochemically interacted with both electrodes by heterogeneously doped nitrogen species. The NMTiO_2_ with rich nitrogen and mesopore cages was used as the anode and coupled with the cathode made of NG nanosheets. The weight ratio of electrode materials for anode and cathode was optimized as *m*_anode_:*m*_cathode_ = 1:3.5 by matching charge balance. Figure 4b,d displays the CV curves for anode and cathode half‐cells as well as the assembled full cell at the scan rate of 1 mV s^−1^. The curves for anode and cathode with distinct redox peaks could be determined. The CV curves for the full cells demonstrate the charge storage mechanism attributed to the combination of Faradaic and non‐Faradaic reactions. The energy storage mechanisms have also been explored by the galvanostatic charging/discharging profiles of anode and cathode electrodes (Figure 4c). The NMTiO_2_ anode shows the Faradaic reaction curve for Na‐ion insertion/desertion, while the NG cathode gives the capacitive reaction curve. While symmetric NG//NG full‐cell devices using organic electrolytes have the limited operation potential of 2.5 V due to the electrolyte decomposition,^38^ the operating potential of 1–3.8 V for the assembled NMTiO_2_//NG full cell is determined to allow higher energy density and stable operation. Figure 4e shows the galvanostatic charge–discharge profiles of full cells at different current densities. The linear curves of voltage profiles indicate typical capacitive behaviors during charging and discharging reactions. The calculated performance (Figure 4f) shows a maximum energy density of 90.5 Wh kg^−1^ at the power density of 91.1 W kg^−1^. Also, it gives the high power density up to 25 920 W kg^−1^ at a condition of ≈20 C (7 A g^−1^) outperforming that of a conventional sodium‐ion battery by more than 400‐folds. The results clarify that the NMTiO_2_//NG full cells outperform the other full‐cell devices of same materials such as TiO_2_//NaLi_0.2_Ni_0.25_Mn_0.75_O,^39^ Na_2_Ti_6_O_13_//Na_3_V_2_(PO_4_)_2_F_3_,^40^ NaTi_2_(PO_4_)_3_//Na_3_V_2_(PO_4_)F_3_,^41^ Li_4_Ti_5_O_12_//Na_3_V_2_(PO_3_)_3_,^42^ Na_2_Ti_3_O_7_//Na_2/3_(Ni_1/3_Mn_2/3_)O_2_,^43^ and Na_2_Ti_3_O_7_//GF (graphene oxide (GO) film)^44^ in the Ragone plots, demonstrating the exceptional energy and power energy densities. In addition, the highest energy density of the asymmetric NMTiO_2_//NG full cell exceeds that of 25.6 Wh kg^−1^ for the symmetric NG//NG full cell by more than threefold (Figure 4f; Figure S14, Supporting Information). Moreover, the hybrid Na‐ion energy storage is determined to act as a bridge between batteries and supercapacitors so that they fill the gaps in the field of current energy storage systems. Furthermore, the NMTiO_2_//NG full‐cell device leads to an excellent stability over 10 000 cycles at a current density of 3 A g^−1^ (≈10 C) with the high capacity retention and nearly 100% coulombic efficiency (Figure 4g; Figure S15c, Supporting Information). The full‐cell devices with high mass loadings (Figure S15a,b, Supporting Information) exhibit high capacities without critical capacity loss. In addition, although the pore and nanoparticle size were increased and the pore distribution number decreased, we find that the crystallinity and porosity of NMTiO_2_ after 10 000 cycles were swell maintained (Figure S16, Supporting Information). Additionally, we demonstrated the photovoltaic charging module and USB chargeable light emitting display (LED) device (Figure 4f, inset; Figure S17 and Videos S1 and S2, Supporting Information) integrating full‐cell devices. Obviously, these prototype devices have allowed to charge full‐cell devices very quickly charged within ≈20 s.

**Figure 4:**
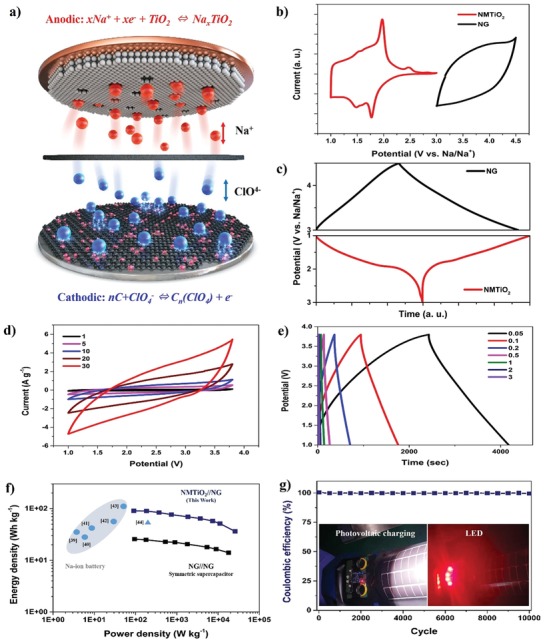
The electrochemical performances of full cells. a) The full‐cell configuration illustrates that Na^+^ and ClO^4−^ ions in the electrolyte have electrochemical interactions with both the anode structure having mesoporous channels and also the cathode structure having heterogeneously doped nitrogen species. b) The CV profiles of half‐cells prepared from NMTiO_2_ anode and NG cathode materials. c) The charge–discharge profiles of NMTiO_2_ and NG in half‐cell configurations. d) The CV profiles of the NMTiO_2_//NG full cells on the various scan rates (mV s^−1^). e) The charge–discharge profiles on different current densities (A g^−1^). f) The Ragone plot (refs: TiO_2_//NaLi_0.2_Ni_0.25_Mn_0.75_O*_δ_*,^39^ Na_2_Ti_6_O_13_//Na_3_V_2_(PO_4_)_2_F_3_,^40^ NaTi_2_(PO_4_)_3_//Na_3_V_2_(PO_4_)F_3_,^41^ Li_4_Ti_5_O_12_//Na_3_V_2_(PO_3_)_3_,^42^ Na_2_Ti_3_O_7_//Na_2/3_(Ni_1/3_Mn_2/3_)O_2_,^43^ and Na_2_Ti_3_O_7_//GF^44^). g) The capacity retention (inset: demonstrated photovoltaic charging and LED modules).

In summary, this work demonstrates the high‐performance electrochemical storages using the 3D nitrogen‐doped mesoporous structures synthesized through the solvothermal pyrolysis of an MOF at a low temperature. Rich open mesopore channels and heterogeneous dopants introduced in the NMTiO_2_ architecture are on distinct advantages. Interestingly, this NMTiO_2_ allowed stable cycling performance over long charge–discharge cycles and high capacity retention even at an extremely high current. In addition, the hybrid NMTiO_2_//NG full‐cell device was demonstrated to give the high energy density superior to that of a symmetric device by more than threefold, the high power density of 25 920 W kg^−1^ exceeding that of a typical sodium‐ion battery by several hundred fold, and robust cycle stability over 10 000 charge–discharge cycles with excellent capacity retention. Moreover, the NMTiO_2_//NG full‐cell devices were demonstrated to be chargeable within a few tens of seconds by the USB LED charger and flexible photovoltaic charging module. These results support that the hybrid sodium‐ion energy storage full cells assembled with nitrogen‐doped mesoporous anode and nitrogen‐doped graphene cathode electrodes provide rich active sites and rapid transport pathways for electrons and ions during repeated charging–discharging cycles, so that they show remarkably high energy density, ultrafast charging capability on excellent power density, and robust cycle stability over a long cycle life. Consequently, we expect that our findings provide a new solution to realize high‐performance electrode materials from metal–organic frameworks, thus, adaptable to develop a diverse range of metal oxides usable for high‐performance energy storage devices.

## Experimental Section

##### Synthesis of NH_2_‐MIL‐125(Ti)

Titanium isopropoxide (0.6 mmol) and 2‐aminoterephthalic acid (1.2 mmol, H_2_BDC‐NH_2_) were dissolved in a mixed solvent of *N*,*N*‐dimethylformarmide and methanol (20 mL, DMF:MeOH = 1:1 in volume), and then put into a 35 mL glass tube, which was capped by a rubber septum and placed in a microwave oven (Discover S‐class, CEM). The mixture was heated to 150 °C, held for 1 h, and then cooled to room temperature. The yellow powder product was separated by centrifugation. After washing with DMF several times, the product was stored in methanol. The product was dried and evacuated in vacuum oven at 60 °C for 1 day.

##### Pyrolytic Conversion of NH_2_‐MIL‐125 (Ti) to NRTiO_2_

The powder products of the NH_2_‐MIL‐125 (Ti) were placed in an alumina boat, placed within box furnace with a fixed air flow. Under the steady air flow, the samples could be decomposed by thermal oxidation reaction. The sample was annealed to 350 °C, kept for 2 h, and then cooled to room temperature. The product appeared as a light‐yellow powder. In order to obtain a highly porous structure, this annealing procedure was conducted at a relatively low temperature and long reaction time considering the crystallization temperature.

##### Synthesis of NG

The NG was prepared via the nitrogen plasma treatment of a reduced graphene oxide (RGO), where GO nanosheets were synthesized by Hummers' method. The graphite flake (5 g) and sodium nitrate (2.5 g, NaNO_3_) were dissolved in concentrated sulfuric acid (120 mL, H_2_SO_4_), and the mixture was stirred during 20 min. Then, potassium permanganate (15 g, KMnO_4_) was slowly added into the mixture in the ice bath blow 20 °C, where it was maintained for 15 min, and then it was stirred for 4 h under 40 °C. After then, deionized water (100 mL) was slowly added into the mixture, where it was maintained for 1 h. The mixture was cooled to room temperature and hydrogen peroxide (2 mL, H_2_O_2_) was added into the mixture. The mixture was washed via vacuum filtration with hydrochloric acid (HCl), acetone, and DI water, respectively. The product was dried via freeze drying for further use. Finally, the GO solution (1 mg mL^−1^) was chemically reduced with hydrazine (1 µg mL^−1^) as a reduction agent in oil bath at 80 °C. Then, the product was filtered and dried following the same method as for a GO product. The RGO solution was drop‐casted onto a glass, and put into plasma‐enhanced chemical vapor deposition (PECVD). Then, hydrogen and nitrogen gas plasmas were sequentially applied for 3 and 10 min, respectively.

##### Characterization

The morphology and structures of the samples were analyzed by the field emission SEM (Hitachi, SU‐5000) and the high‐resolution STEM–EDS (JEM, ARM200F, Cs‐corrected STEM). The TEM specimens were prepared by dropping the dispersed samples in volatile solvents on the TEM grid (Ted Pella Inc.) The X‐ray diffraction data were collected using a SmartLab θ–2θ diffractometer in reflectance Bragg–Brentano geometry. The diffractometer used a Johansson‐type Ge(111) monochromator filtering Cu Kα1 radiation at 1200 W (40 kV, 30 mA) to minimize line broadening and was equipped with a high‐speed 1D detector (D/teX Ultra). The 2θ range was 3°–90°. The surface chemical states were investigated by the XPS (Thermo VG Scientific Sigma Probe) analysis. Moreover, the porosity was analyzed through N_2_ adsorption/desorption isotherm measurements at 77 K using the Brunauer–Emmett–Teller (Quantachrome Qudrasorb‐evo). The chemical bonding information for the functional groups present in samples was also elucidated by using an FTIR spectroscopy (FT/IR‐6100, JASCO) analysis. The NMTiO_2_ and reference samples were ground with KBr using a mortar and a pestle in the ratio of 1:100 in weight, and then the mixture was pressurized through the hand‐operated pressurization to the thin pellet having the width of 6 mm. The spectra were obtained at 2 cm^−1^ with 50 scans per spectrum in the range of 500–4000 cm^−1^. The thermal behavior of NH_2_‐MIL‐125 (Ti) during the thermal oxidation procedure was investigated by thermogravimetric analysis (NETZCH TG 209 F1 Libra) in a range of 20–700 °C under 5 °C min^−1^ heating rate and air flow conditions. To investigate the changes on nitrogen doping effects, the diffused absorption spectra were obtained by a VARIAN Cary‐300 UV–vis spectrophotometer using powder samples as prepared.

##### Electrochemical Characterization

The electrochemical properties of the NMTiO_2_ and NG were characterized by using the 2032‐type coin cells in which Celgard 2400 and Na foil were used as separators and counter/reference electrodes, respectively. On the sample preparation, the active material, super P, and polyvinylidene fluoride (PVDF) (80:10:10 in weight) were dispersed in *N*‐methyl‐2‐pyroolidinone (NMP) to form a slurry. Then, the slurry was cast onto the Cu or Al foil using the doctor blade technique. The cast electrodes were dried in a vacuum oven at 80 °C overnight. The standard organic electrolyte was used in which the 1 m NaClO_4_ was dissolved in ethylene carbonate (EC) and diethyl carbonate (DEC) (EC:DEC = 1:1 in volume). The entire cell preparation steps were conducted in an argon‐filled glove box with the moisture content and oxygen levels less than 1 ppm. All of the electrochemical performance measurements were done at room temperature using a potentiostat/galvanostat/EIS system (VSP, Bio‐Logic). Moreover, electrochemical analyses of the half‐cells were measured in an operating potential range of 1–3 V (anode) and 3–4.5 V (cathode) versus Na/Na^+^, respectively. The impedance analysis was conducted in a frequency range from 0.01 Hz to 1000 kHz with the amplitude of 5 mV. The GITT measurements were carried out at ≈0.1 C in a period of 1 min. Before the measurements, the pre‐sodiation procedure was conducted by attaching the NMTiO_2_ electrode with the Na foil during 1 h. The mass loading was measured by the ultra‐microbalance (XP2U, Mettler Toledo, *d* = 0.1 µg). The electrochemical performances of full‐cell devices were determined at an average mass loading amount of 3–4.5 mg cm^−2^ for the total mass of anode and cathode materials.

##### Assembly and Electrochemical Measurements of Full‐Cell Devices

Sodium‐ion energy storage full cells were assembled using the NMTiO_2_ composite as the anode and the NG as the cathode, respectively. The NG cathode was prepared in the same manner as the anode. The electrochemical analysis was carried out using the same method as in the half‐cell. The electrochemical measurements of the NMTiO_2_//NG full cells were carried out in an operating potential range of 1–3.8 V. The electrochemical performances of full‐cell devices were determined on the mass loading of 3–12 mg cm^−2^ for the total mass of anode and cathode electrodes. The overall cell energy/power densities were calculated based on the total mass of cathode and anode materials (Section S3, Supporting Information).

## Conflict of Interest

The authors declare no conflict of interest.

## Supporting information

Supporting Information

Supplemental Video 1

Supplemental Video 2
