# Supplementary material for: Synthesis of Nitrogen‐Doped Mesoporous Structures from Metal–Organic Frameworks and Their Utilization Enabling High Performances in Hybrid Sodium‐Ion Energy Storages
Source: Adv Sci (Weinh). 2020 Jan 27;7(6):1902986. doi: 10.1002/advs.201902986 (PMC7080513; doi:10.1002/advs.201902986)
Supplement: Supplementary file 1 — Supporting Information [file ADVS-7-1902986-s001.pdf]

Copyright WILEY-VCH Verlag GmbH & Co. KGaA, 69469 Weinheim, Germany, 2020.

ADVANCED SCIENCE
Open Access

Supporting Information

for *Adv. Sci.,* DOI: 10.1002/advs.201902986

Synthesis of Nitrogen-Doped Mesoporous Structures from Metal–Organic Frameworks and Their Utilization Enabling High Performances in Hybrid Sodium-Ion Energy Storages

*Gyu Heon Lee and Jeung Ku Kang**

WILEY-VCH

Copyright WILEY-VCH Verlag GmbH & Co. KGaA, 69469 Weinheim, Germany, 2013

Supporting Information

Synthesis of nitrogen-doped mesoporous structures from metal-organic frameworks and their utilization to enable high performances in hybrid sodium-ion energy storages

*Gyu Heon Lee and Jeung Ku Kang**

G. H. Lee, Prof. J. K. Kang
Department of Materials Science and Engineering
Korea Advanced Institute of Science and Technology (KAIST)
291 Daehak-ro, Yuseong-gu, Daejeon 34141, Republic of Korea
*Email: jeungku@kaist.ac.kr

Prof. J. K. Kang
Graduated School of Energy, Environment, Water, and Sustainability (EEWS)
NanoCentury KAIST Institute
Korea Advanced Institute of Science and Technology (KAIST)
291 Daehak-ro, Yuseong-gu, Daejeon 34141, Republic of Korea

Section S1. Method details

S1.1. Synthesis of NH_2-MIL-125(Ti)

Titanium isopropoxide (0.6 mmol) and 2-aminoterephthalic acid (1.2 mmol, $H_2BDC-NH_2$) were dissolved in a mixed solvent of N, N-dimethylformarmide and methanol (20ml, DMF:MeOH = 1:1 in volume), and then put into a 35 mL glass tube, which was capped by a rubber septum and placed in a microwave oven (Discover S-class, CEM). The mixture was heated to 150 °C, held for 1 h, and then cooled to room temperature. The yellow powder product was separated by centrifugation. After washing with DMF several times, the product was stored in methanol. The product was dried and evacuated in vacuum oven at 60 °C for 1 day.

S1.2. Pyrolytic conversion of NH_2-MIL-125(Ti) to $NMTiO_2$

The powder products of the NH_2-MIL-125 (Ti) were placed in an alumina boat, placed within box furnace with a fixed air flow. Under the steady air flow, the samples could be decomposed by thermal oxidation reaction. The sample was annealed to 350 °C, kept for 2 h, and then cooled to room temperature. The product appeared as a light-yellow powder. In order to obtain highly porous structure, this annealing procedure was conducted at a relatively low temperature and long reaction time considering the crystallization temperature.

S1.3. Synthesis of RGO

The NG (nitrogen-doped graphene) was prepared via the nitrogen plasma treatment of a reduced graphene oxide (RGO), where the GO nanosheets were synthesized by Hummers' method.[S1] The graphite flake (5 g) and sodium nitrate (2.5 g, $NaNO_3$) were dissolved in concentrated sulfuric acid (120 ml, H_2SO_4), and the mixture was stirred during 20 min. Then, potassium permanganate (15 g, $KMnO_4$) was slowly added into the mixture in ice bath blow

20 °C, where is was maintained for 15 m, and then it was stirred for 4 h under 40 °C. After then, DI water (100 ml) was slowly added into the mixture, where it was maintained for 1 h. The mixture was cooled to room temperature and the hydrogen peroxide (2 ml, H_2O_2) was added into the mixture. The mixture was washed via vacuum filtration with Hydrochloric acid (HCl), Acetone and DI water, respectively. The product was dried via freeze drying for further use. Finally, the GO solution (1mg/ml) was chemically reduced with hydrazine (1µg/ml) as a reduction agent in oil bath at 80 °C. Then, the product was filtered and dried following the same method as for a GO product.

S1.4. Synthesis of NG

The RGO solution was drop casted onto a glass, and put into the plasma enhanced chemical vapor deposition (PECVD) chamber. Then, hydrogen and nitrogen gas plasma were sequentially applied for 3m and 10 m, respectively.

Section S2. Structural analysis details

S2.1. TEM (Transmission electron microscopy)

For the TEM observation (Tecnai F20 produced by Philips / JEM-ARM200F produced by JEOL / Tiatan cubed G2 produced by FEI company), the samples were dispersed with the acetone solvent and put on a Cu mesh grid. An energy dispersive spectrometer (EDS) attached to the TEM was used to obtain the local elemental information, the line elemental profile and the elemental mapping.

S2.2. SEM (Scanning electron microscope)

For the SEM observation ((Hitachi, SU-5000), the samples were dispersed with the acetone solvent and dropped on a small piece of a silicon wafer.

S2.3. XPS (X-ray photoelectron spectroscopy)

The XPS spectra were obtained using a Sigma Probe of Thermo VG Scientific, which is equipped by a 350 W Al anode x-ray source along with a multi-anode, a pulse counting, and a hemispherical analyzer. The spectra were collected using an incident photon energy of 1486.6 eV and were also corrected for the detector's work function.

S2.4. XRD (X-ray diffraction spectroscopy)

The powder X-ray data were collected using a SmartLab θ-2θ diffractometer in the reflectance Bragg-Brentano geometry employing a Johansson type Ge (111) monochromator filtered Cu Kα1 radiation at the 1200W (40 KV, 30 mA) power and equipped with a high speed 1D detector (D/teX Ultra). The powders of the sample structures were held in a holder stage and scanned by the scan speed of 2 °/min in a continuous mode.

S2.5. FT-IR (Fourier Transport-Infrared Spectroscopy)

The chemical bonding information of the functional groups present in samples was analyzed by using a FTIR spectroscopy (FT/IR-6100, JASCO). The $NMTiO_2$ and reference samples were ground with KBr using mortar and pestle in the ratio of 1:100 in weight and then the mixture was pressurized by the hand-operated pressure to the thin pellet with an width of 6 mm. The spectra were obtained at 2 cm^{-1} with 50 scans per spectra in the range of 500 to 4000 cm^{-1}.

S2.6. Surface area and pore size analyzer

The N_2 adsorption and desorption isotherms were determined by a Quantachrome Instruments Autosorb-1c apparatus at 77 K. The samples were outgassed at 333 K and for 24 hours before measurements.

S2.7. Thermal oxidation behavior analysis.

The thermal behavior of NH2-MIL-125 (Ti) during the thermal oxidation procedure was investigated by thermogravimetric analysis (NETZCH TG 209 F1 Libra) in a range of 20 to 700 ℃ under 5 ℃ per min heating rate and air flow conditions.

S2.8. UV-visable absorption spectra.

To investigate the changes on nitrogen doping effects, the diffused apsorption spectra were optained by a VARIAN Cary-300 UVVis spectrophotometer using powder samples as prepared.

Section S3. Electrochemical characterization details

S3.1. Electrochemical half cells

The electrochemical properties of the NMTiO_2 were characterized by using the 2032 type coin cells in which Celgard 2400 and Na foil were used as separators and counter/reference electrodes, respectively. On the sample preparation, the active material, super P, and polyvinylidene fluoride (PVDF) (80:10:10 in weight) were dispersed in N-methyl-2-pyroolidinone (NMP) to form a slurry. Then, the slurry was cast onto the Cu or Al foil using the doctor blade technique. The cast electrodes were dried in a vacuum oven at 80 ˚C overnight. We used the standard organic electrolyte in which the 1M $NaClO_4$ is dissolved in ethylene carbonate (EC) and diethyl carbonate (DEC) (EC : DEC = 1 : 1 in volume). The entire cell preparation steps were conducted in an argon-filled glove box with the moisture content and oxygen levels less than 1 ppm. All of the electrochemical performance measurements were done at room temperature using a potentiostat/galvanostat/EIS system (VSP, Bio-Logic). Moreover, electrochemical analyses of the half-cells were measured in an operating potential range of 1 to 3 V (anode) and 3 to 4.5 V (cathode) versus. Na/Na^+,

respectively. The impedance analysis was conducted in a frequency range of 0.01 Hz to 1000 kHz with the amplitude of 5 mV. Before the measurements, the pre-sodiation procedure was conducted by attaching the $NMTiO_2$ electrode with the Na foil during 1 hour. The GITT measurements were carried out at ~0.1 C in a period of 1 minute. The mass loading was measured by the ultra-microbalance (XP2U, Mettler Toledo, d= 0.1 μg).

S3.2. Hybrid full cells

Hybrid sodium-ion energy storage full cell devices were assembled using the $NMTiO_2$ composite as the anode and the NG as the cathode, respectively. The NG cathode was prepared in the same manner as the anode. All the electrochemical analyses were performed using the same methods used in the half cell. The electrochemical measurements of the $NMTiO_2$//NG full-cells were carried out in an operating potential range of 1 to 3.8 V. The electrochemical performances of full cell devices were determined on the mass loading of 3 to 12 mg cm^{-2} for the total mass of anode and cathode electrodes. The overall cell energy/power densities were calculated based on the total active mass of cathode and anode materials. The specific capacitance (C_S) is calculated by using the following equations[S2] of

$C_{cell} = i \times t / \Delta V$

$C_s = 4\ C_{cell} / m$

where i is the applied current (A), t is the discharge time (s), m is the total mass (g) of active materials in both the anode and cathode, and ΔV is the potential difference (V). The power density (P, W kg^{-1}) and the energy density (E, Wh kg^{-1}) were calculated using the following equations[S3], as described by

$P = V \times i / m$

$E = P \times t / 3600$

$V = (V_{max} + V_{min}) / 2$

where V_{max} and V_{min} are the potentials at the beginning and the end of the discharge (V).

Section 4. Experimental characterization details

S4.1 Effects on the nitrogen doping of $NMTiO_2$

We investigated the color changes on nitrogen-doped TiO_2 NPs by UV-vis absorption spectroscopy as shown in Supplementary Figure S1a. The shift in absoprtion specra is indicated by the nitrogen-doping on balance bands. We also found that the bandgap was reduced by about 0.54 eV. Depending on this band structure difference, the $NMTiO_2$ sample shows a light-yellow color (Supplementary Figure S1b).

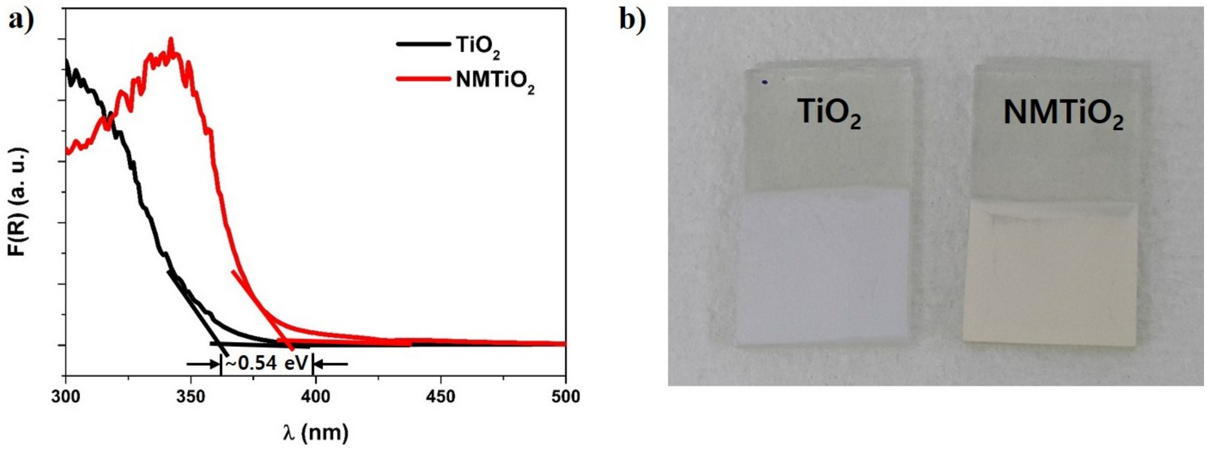

Figure S1. UV-vis absorption spectra and real photo images. (a) UV-vis absorbansce specctra and (b) real photo images of bare TiO_2 and n-doped TiO_2 samples.

S4.2 Morphology and size of $NMTiO_2$

The morphology and size of NH_2-MIL-125 (Ti) and $NMTiO_2$ samples were observed by the SEM and TEM analyses, as shown in Figs. S1 and S2. The top images correspond to those for the bare NH_2-MIL-125 (Ti). This product appears in the rectangular shape with round edges and its average size is about 1 um width and 0.2 um thickness. Likewise, the size and shape of the $NMTiO_2$ (bottom images in Figures S1 and S2) synthesized from the NH_2-MIL-125 (Ti) are quite similar. We have observed the edge shape sharpened during the annealing procedure. Figure S2 also shows that the width and thickness of the $NMTiO_2$ were shrink to 0.5 um and 0.1 um, respectively. Also, mesopores were determined to be introduced into the interior parts of the products, which play to give the fast transport channels for redox ions.

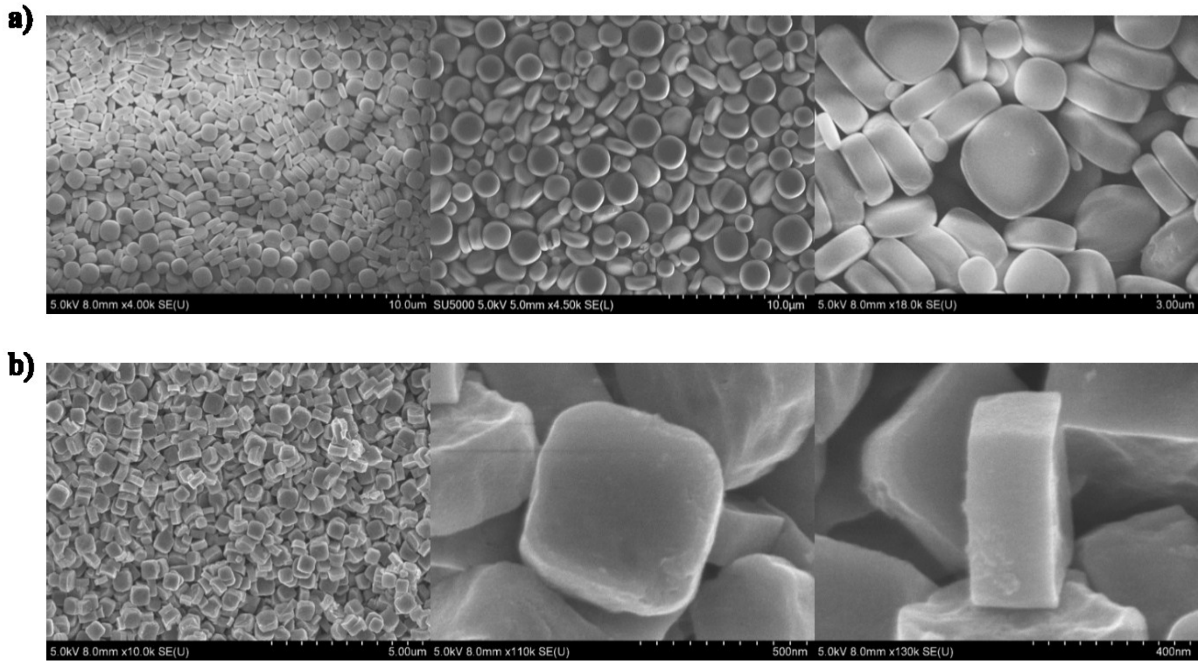

Figure S2. The SEM images. Those for (a) NH_2-MIL-125(Ti) and (b) $NMTiO_2$ at various magnifications.

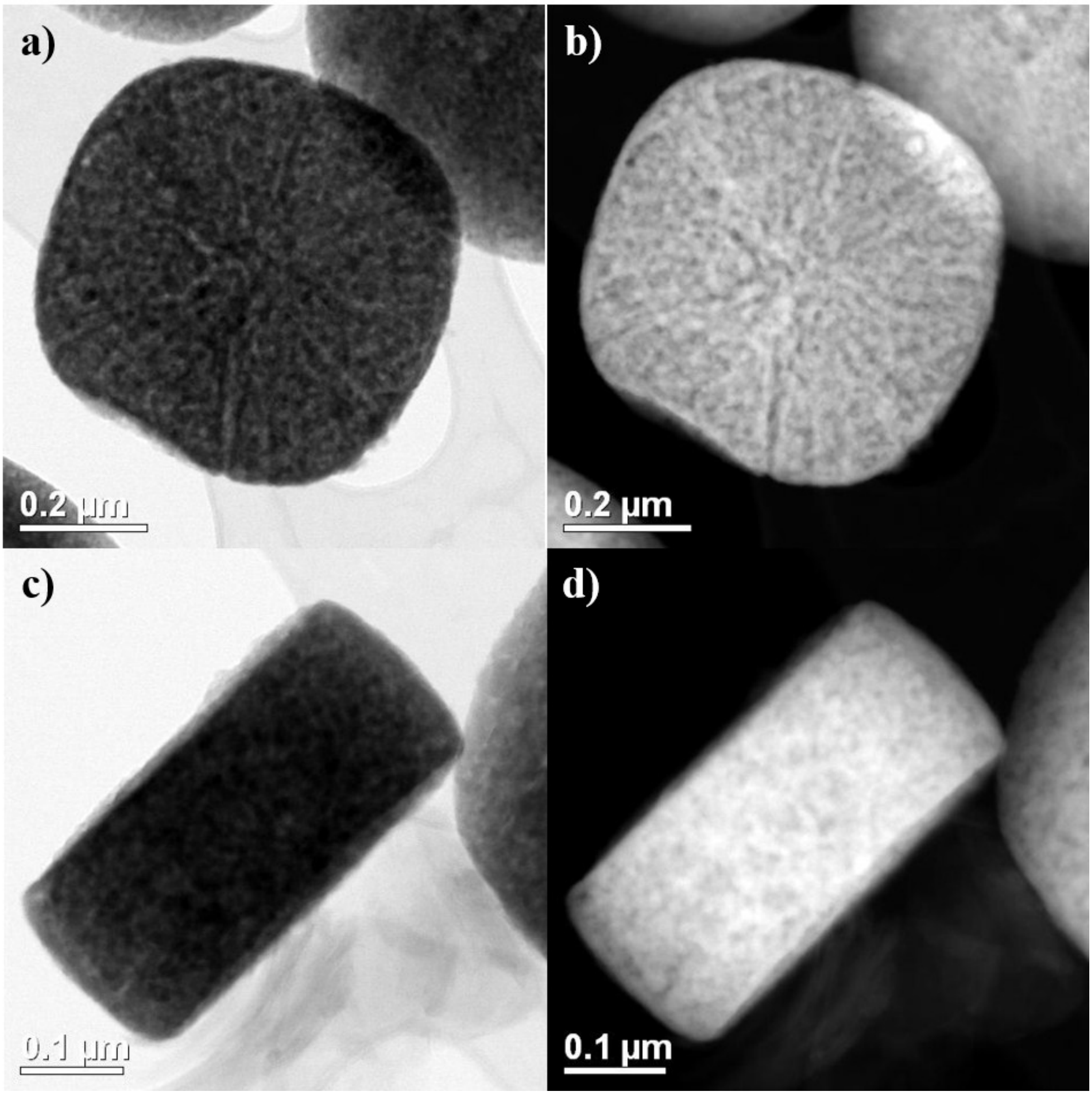

Figure S3. The STEM images of $NMTiO_2$ structures. (a) The brigt field and (b) the dark field images of over views, and c) the bright field and (d) the dark field images of side views.

S4.3. Chemical information of $NMTiO_2$ samples

The constituents of the $NMTiO_2$, which are mainly composed of Ti and O species including the small amount of nitrogen and residual carbon atoms, are confirmed from STEM-EDS, XPS and EELS. As shown in Figure S3, the EDS mapping image presents each element for Ti, O, N, and C atoms.. We also find from the XPS spectra (Figure S4) that the each peak for Ti 2p, O 1s, C 1s, N 1s is detected at the corresponding position.

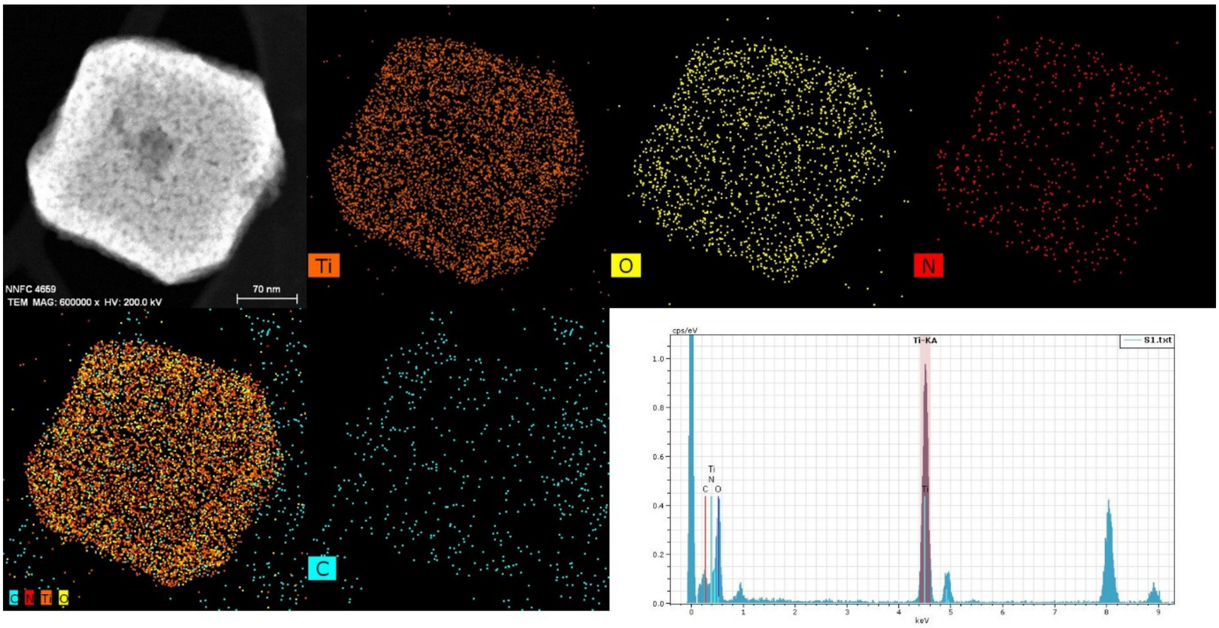

Figure S4. The STEM-EDS images and spectra of $NMTiO_2$ samples.

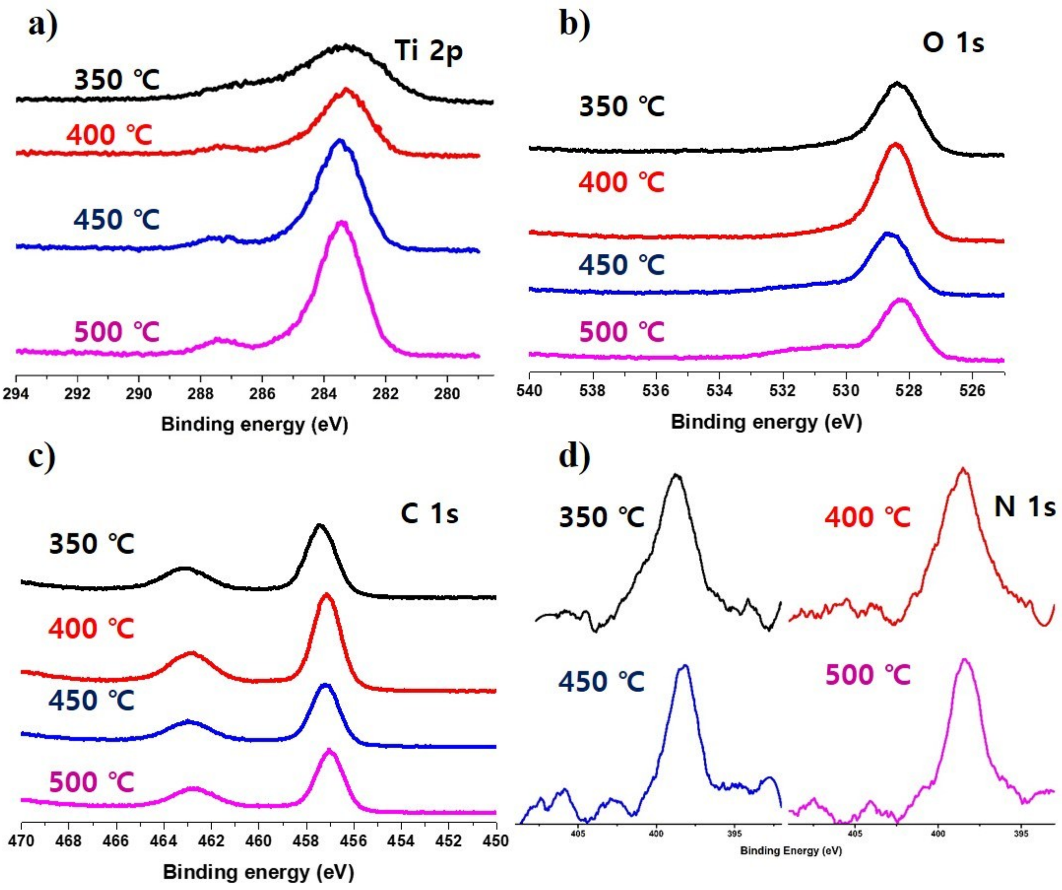

Figure S5. The XPS spectra of $NMTiO_2$ annealed at various temperatures. The obtained peaks of (a) Ti 2p, (b) O 1s, (c) C 1s and (d) N 1s, respectively.

S4.4. Chemical information of NG samples

The chemical information of the nitrogen-doped graphene (NG) is observed from XPS and STEM-EDS ananlyses. As shown in the EDS mapping image (Figure S5), the elements of C, O, N are confirmed and the nitrogen is clearly shown in the mapping image and graph. Also, the XPS analysis (Figure S6) show clearly the peaks for C 1s, O 1s, and N 1s.

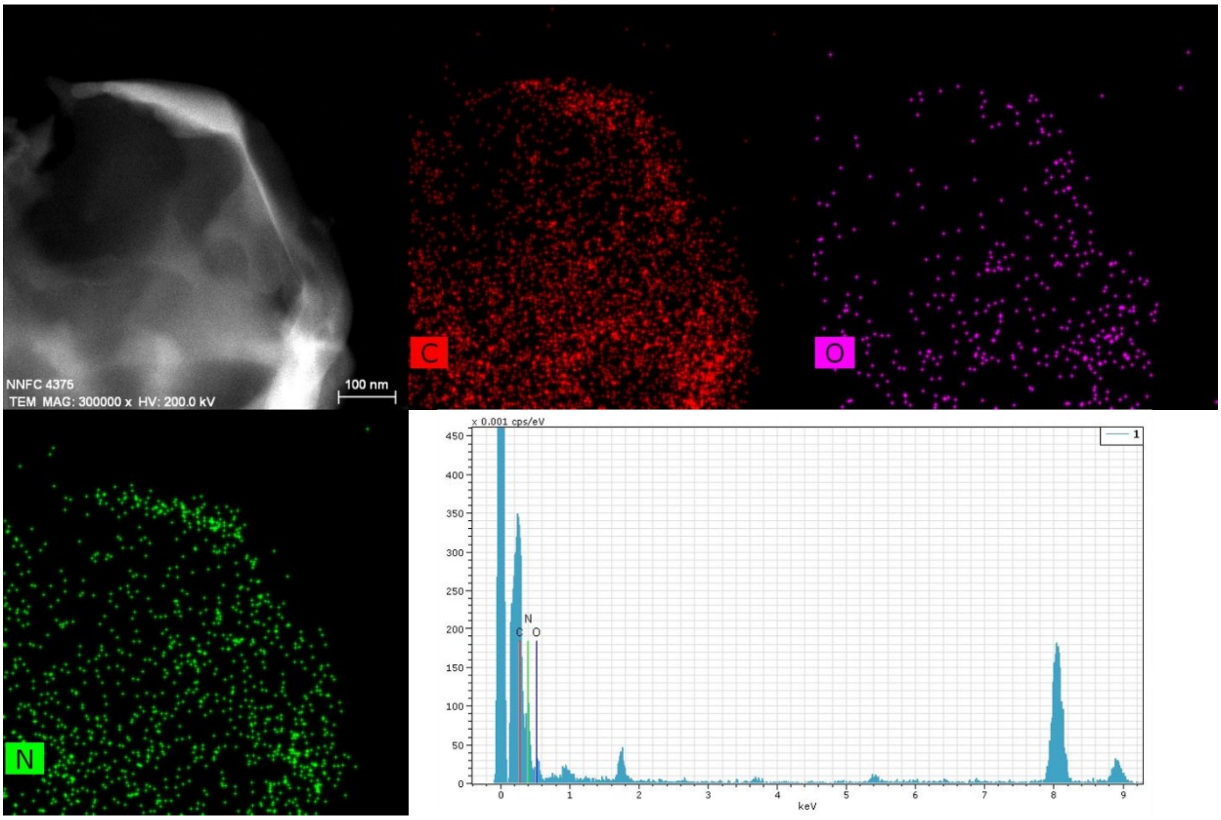

Figure S6. The STEM-EDS images and spectra of NG samples.

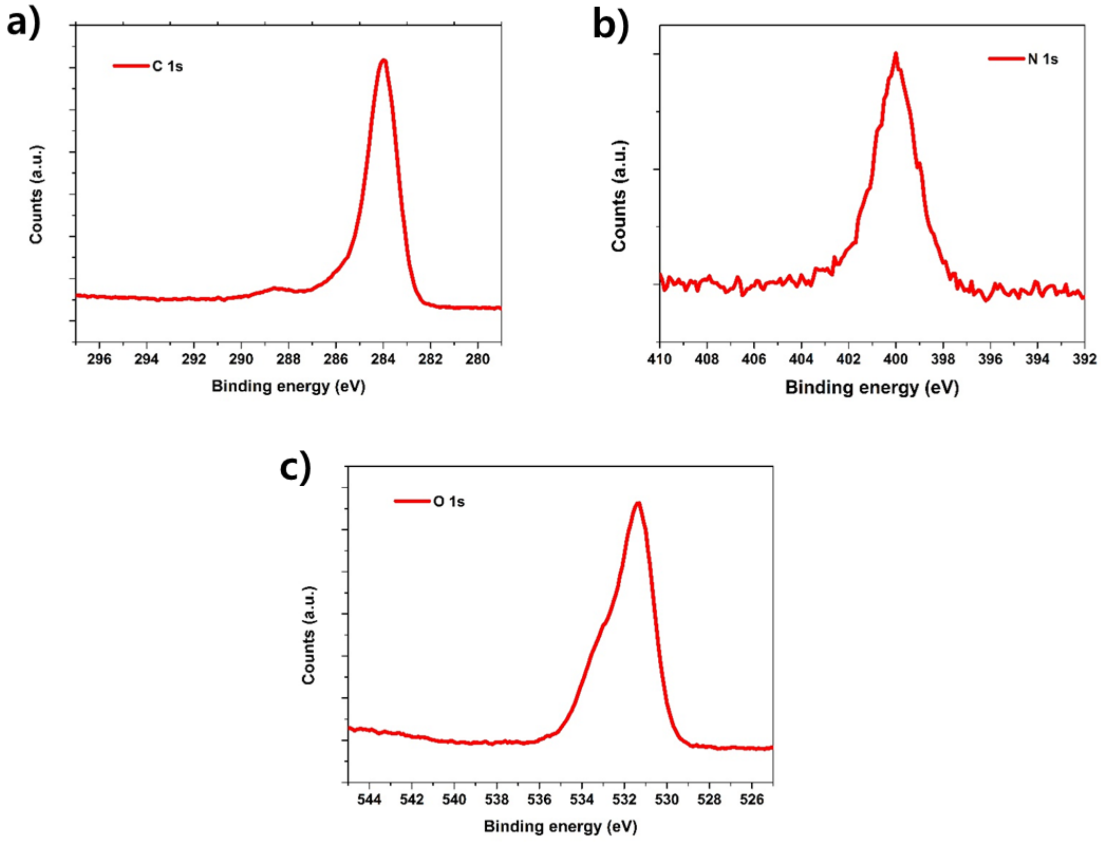

Figure S7. The XPS spectra of NG. The obtained peaks of (a) C 1s, (b) N 1s and (c) O 1s, respectively.

S4.5. Crystal structure and surface area analysis of NH_2-MIL-125 (Ti)

The crystallographic informations on $NMTiO_2$ and NH_2-MIL-125(Ti) were collected through the XRD anaylsis (Figure S7). We find that the diffraction patterns of the $NMTiO_2$ match well with those of the anatase phase (JCPDS card No. 21-1272).[S2] However, the diffraction peaks of the rutile pahse having the broad shape and low intensity have not been deteced. Additionally, the high crystalline NH_2-MIL-125 (Ti) showed the sharp diffraction peaks and the N_2 adsorption/desortion ananlysis demonstrated that the NH_2-MIL-125 (Ti) has a surface area of ~ 1356 m^2 g^{-1} with the typical hysteresis of a microporous material.

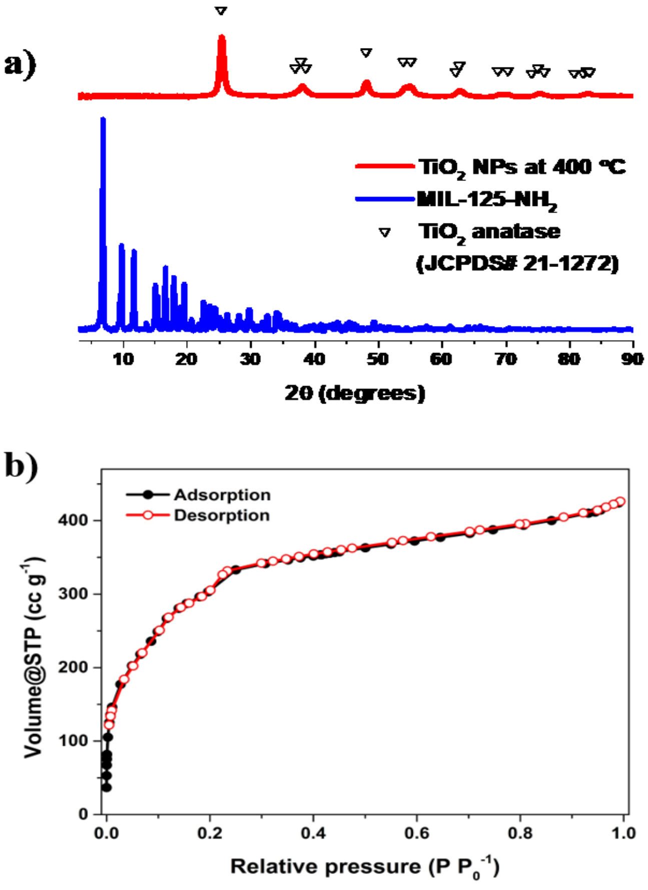

Figure S8. The XRD patterns and N_2 Adsorption/desorption curves. The XRD patterns for (a) NH2-MIL-125 (Ti) and $NMTiO_2$. and (b) the N_2 adsoption/desorption curves of NH_2-MIL-125 (Ti).

S4.6. Chemical binding information for nitrogen dopants in the crystal lattice of $NMTiO_2$

The chemical information of nitrogen species doped in the $NMTiO_2$ crystals was investigated by the FT-IR analysis (Figures 2i and S8). The sample was prepared in the form of a pellet by the pressurization method. The $NMTiO_2$ powder samples were mixed with KBr in the ratio of 1 : 100 in weight in mortar and pestle. Then, the mixture was pressurized by the hand-operated pressurization to the sylinderical and thin pellets. From the investigated results, a variety of the bending and stretching vibration modes for Ti-N, OH, N-H, $NO3^{-1}$, absorbed water molecules and hydroxyl groups were confirmed. These FT-IR and XPS spectra for the nitrogen dopants support that the nitrogen species have been successfully incorporated in the lattice of $NMTiO_2$ nanocrystals.

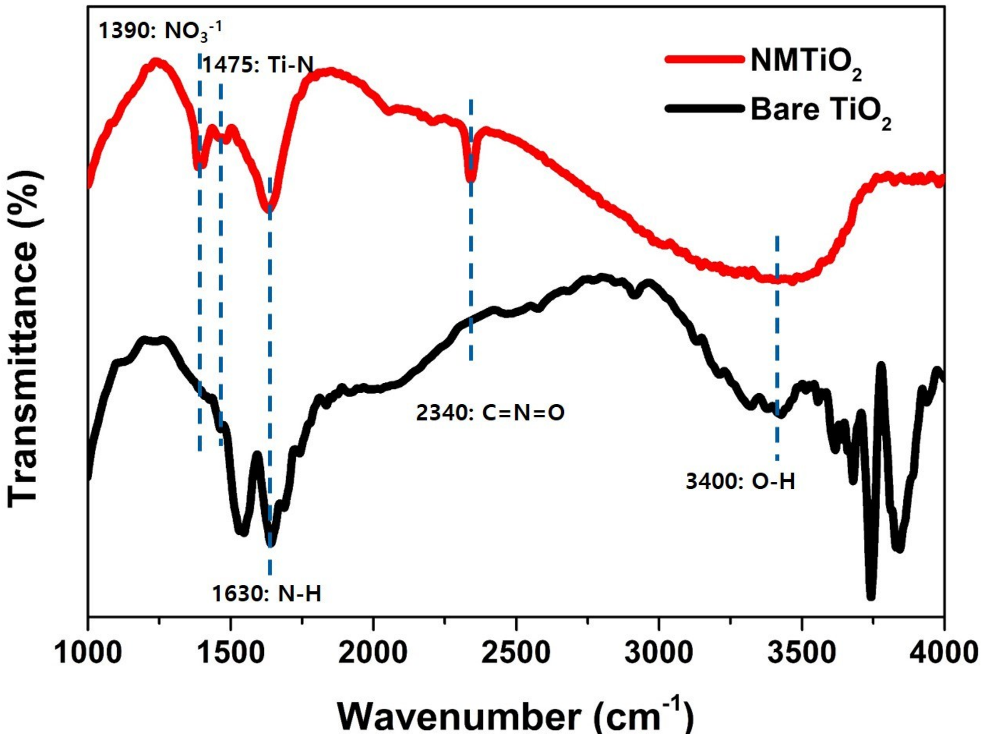

Figure S9. The FT-IR spectra of the $NMTiO_2$ confirming nitrogen dopants in the nanocrystals.

S4.7. Temperature-dependent pore size distribution

The thermal oxidation behavior of NH2-MIL-125 (Ti) under the air flow conditions was analyzed through the thermogravimetric analysis (TGA). In the first range below ~335 ^{o}C, we find that small amounts of surface absorbed water molecules and impurities were removed. After then, the crystallization of titanium ions and the combustion of carbon species were observed to be occurring up to ~ 550 ^{o}C. These results support that the thermal oxidation temperature could be proceeded above 350 ^{o}C.

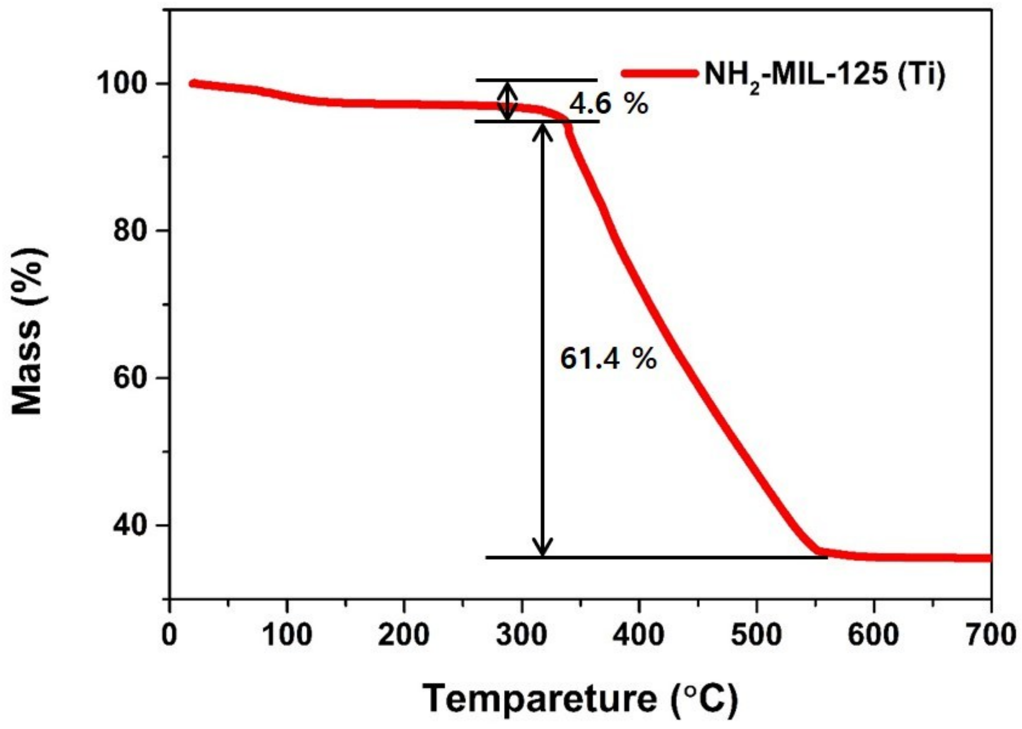

Figure S10. The TG analysis of the NH_2-MIL-125 (Ti) confirming thermal oxidation and crystallization behaviors

S4.8. Temperature-dependent pore size distribution

We studied the temperature-dependent pore size change in the products. In various annealing procedures, we find that the particle and pore size with the pore distribution were clearly changed. The particle and pore size became smaller and the more mesopores were introduced in the products. The pore size analysis (Figure S9) shows clearly that the mesopores with an average size of ~ 4 nm are signicantly increased under the pyrolysis condition at 350 °C.

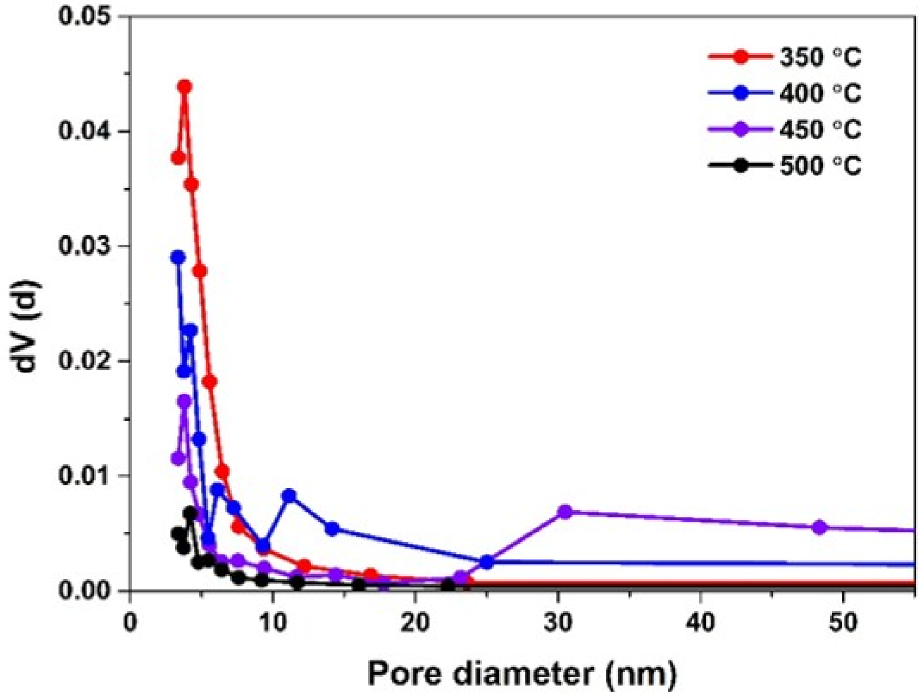

Figure S11. The pore size distribution at different processing temperatures.

S4.9. Temperature-dependent pore size distribution

To understand the diffusion kinetics of sodium-ion in the $NMTiO_2$ anode, the galvanostatic intermittent titration technique (GITT) measurements were analyzed using the $NMTiO_2$ half-cells. Figure S12a shows the time-voltage profile of $NMTiO_2$ during charging/discharging and the voltage plateaus for phase transition by Na^+ insertion were clearly observed during charging and discharging reactions. The difference of discharging behaviors was also shown in Figure S12b and c. The discharging time of the 350 °C sample exhibits to be much longer than that of the 500 °C sample. Moreover, the 350 °C sample was found to accommodate the higher concentration of Na^+ ions up to 1 mole fraction. From the time-voltage profiles of titrations in the voltage plateau region during discharging (Figure S12d and e), we also observed that the 350 °C sample shows the much smaller ΔE_τ of the transient voltage-change during a titration current flux after the relaxation period. It suggests that the 350 °C sample has a relatively higher diffusion coefficient (D_{Na^+}) that can be calculated using the following equation of

$$D_{Na^+} = \frac{4}{\tau}\left(\frac{m_B B_M}{M_B S}\right)^2\left(\frac{\Delta E_S}{\tau(dE_\tau/\sqrt{\tau}}\right)^2 \qquad \left(\tau \ll \frac{L^2}{D_{Na^+}}\right) \quad [S4]$$

where m and M are the mass and the molecular weight of the electrode material, respectively; V_m is the molar volume; S is the active area of the electrode; L is the thickness of the electrode. These results imply that the nitrogen-doped mesoporous TiO_2 synthesized at the low annealing temperature can give the facilitated Na-ion diffusion between the surface and the electrolyte. The plots of potential vesus $\tau^{1/2}$ (Figure S12 f and g) were also found to fit into a straight line.

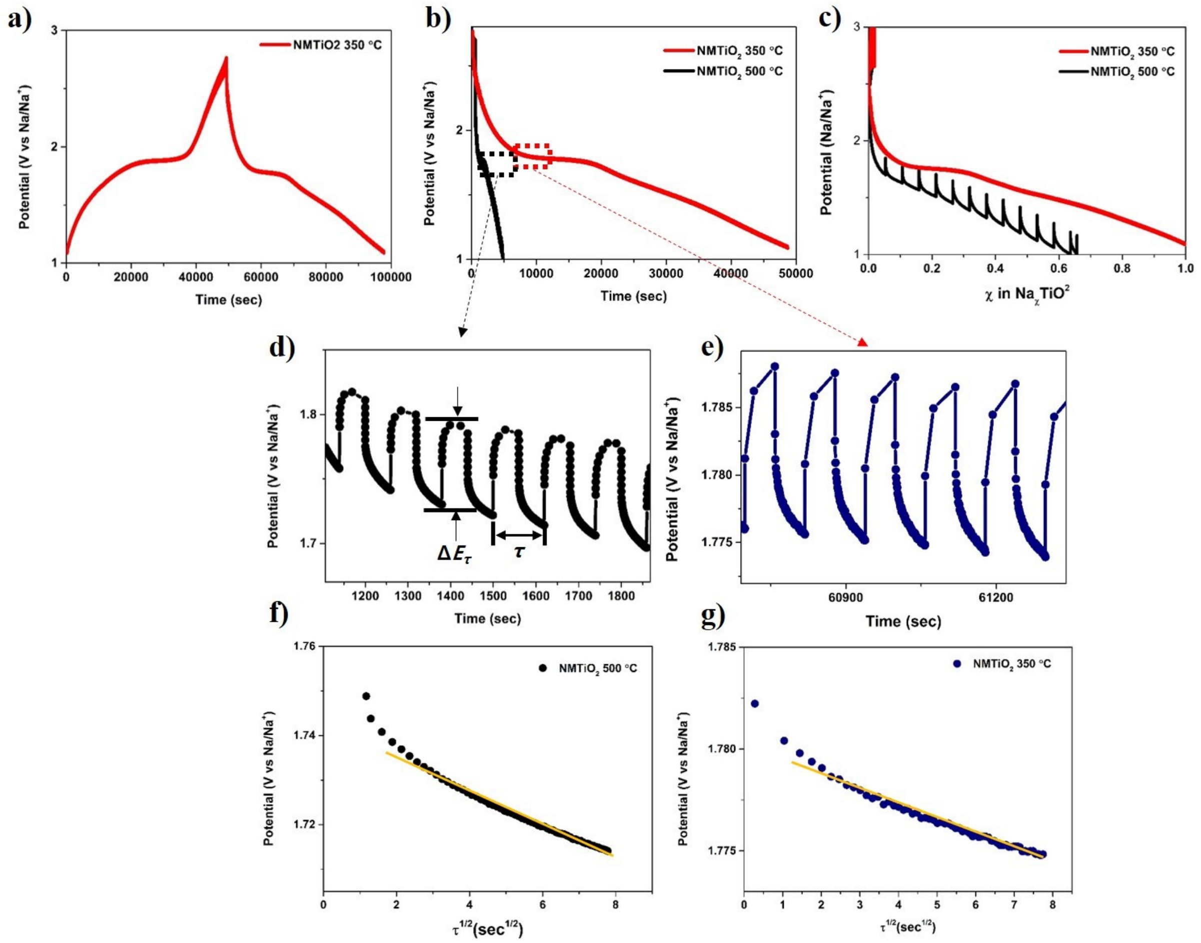

Figure S12. The GITT measurements of $NMTiO_2$ in a half-cell configuration. The time vs. voltage profiles of (a) $NMTiO_2$ 350 ℃ on the charging/discharging processes, (b) $NMTiO_2$ 350 ℃ and 500 ℃ on the discharging process. (c) The composition vs. voltage profiles during discharging. (d) and (e) The time vs. voltage profiles of titrations in the phase transition region. (f) and (g) The relationships of voltage vs. $\tau^{1/2}$.

S4.10. Performance for the NG half cell

The performance for the half-cell of a NG sample as the cathode can be shown in Figure S10. The cathode was fabricated with sodium metal foil as an anode. And also, cathode slurry has a mass ratio of 8:1:1 (NG:PVDF:Super P) and the slurry was coated on aluminum foil by the doctor blading method. The average weight of the total cathode is 2~3.5 mg. The highest capacitance of this electrode is ~78 mAh g^{-1} from the cyclic voltammetry (CV) and galvanic potential measurements. This result represents relatively high capacity.

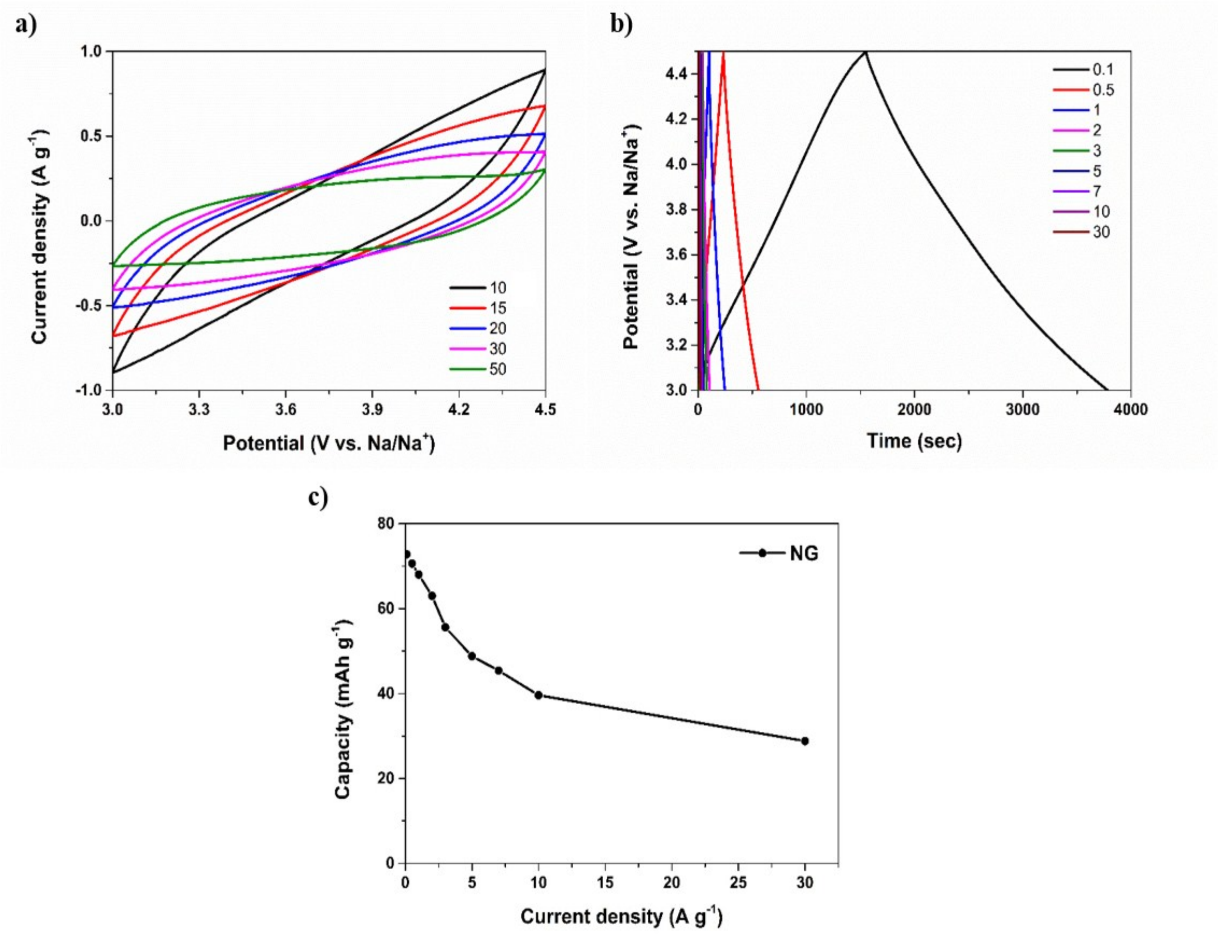

Figure S13. The electrochemical performances for the NG half-cell. (a) The cyclic voltammogram (mV s^{-1}), (b) the charge-discharge profile (A g^{-1}) and (c) the specific capacitance.

S4.11. Performance for the NG//NG full cell

We investigated the performance of the NG//NG full cell device (Figure S11). The anode and cathode electrodes have been also fabricated on copper and aluminum foils as fabricated before. It is notable that the average weight of the total cathode is 4~7 mg. The highest specific capacitance of this full cell is ~ 85.4 F g^{-1} at a current density of 0.1 A g^{-1}, which is determined through the cyclic voltammetry (CV) and galvanic potential measurements.

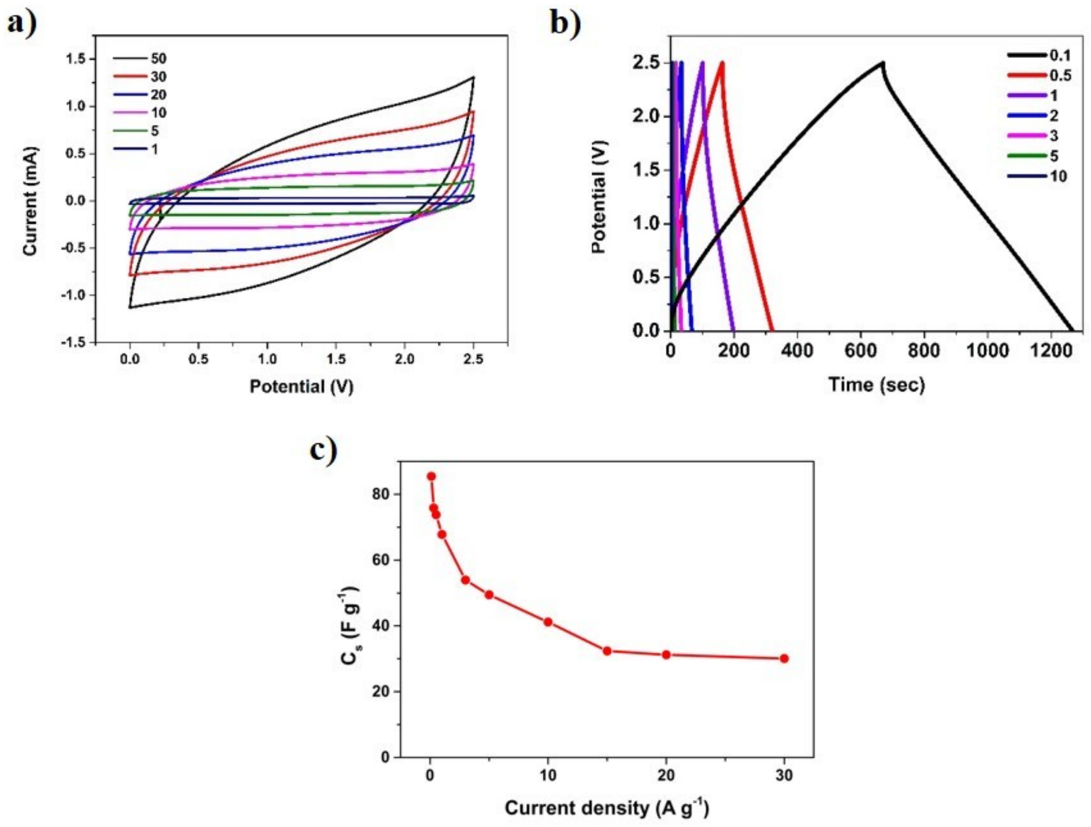

Figure S14. The electrochemical performances for the NG//NG full cell. (a) The cyclic voltammogram (mV s^{-1}), (b) the charge-discharge profiles (A g^{-1}), and (c) the specific capacitance.

S4.12. Cycle performance and capacities of full cell devices with with high mass loadings

We find that the delivered capacities of the full cell devices with high mass loadings exhibit stable performance without large capacity loss. In addition, we carried out the analysis for the stability of the full cell energy storage. The $NMTiO_2$//NG full cell device exhibits an excellent stability over 10,000 cycles at a current density of 3 A g^{-1} with the high capacity retention and nearly 100% coulombic efficiency.

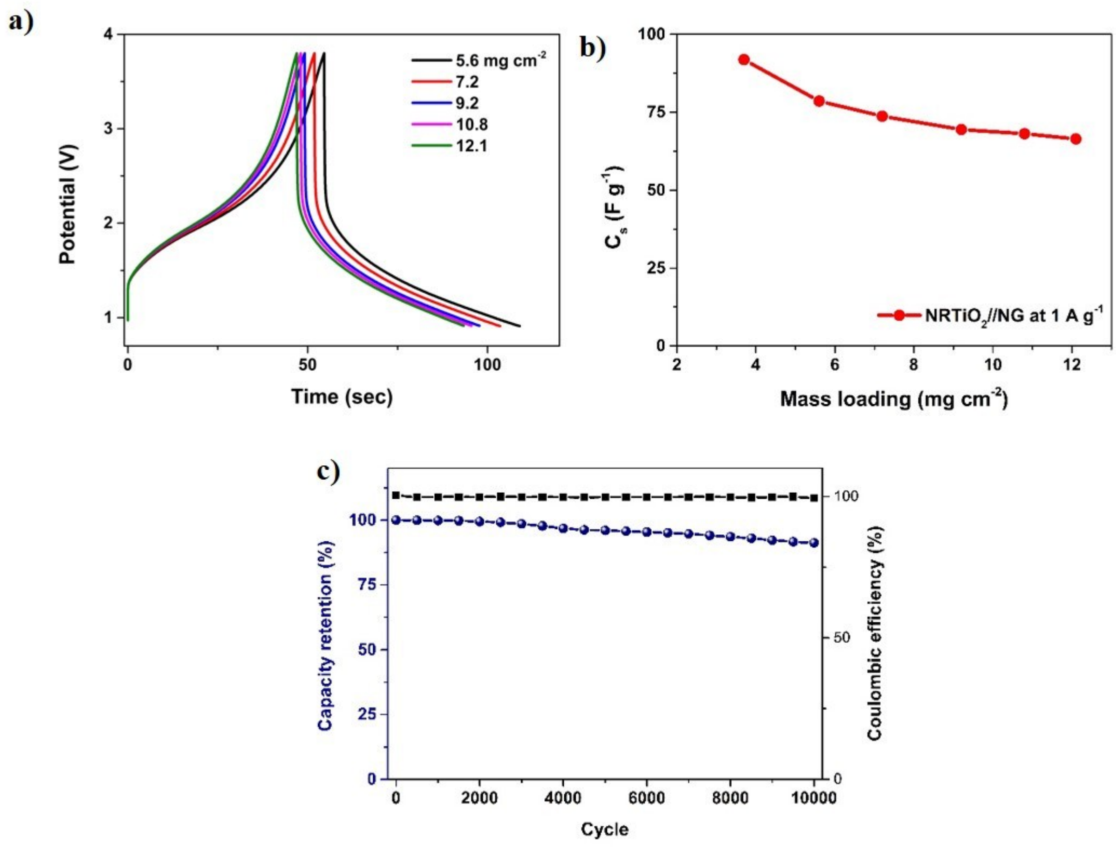

Figure S15. The high mass loading charge-discharge profiles and specific capacities along with cycle stability. a) The charge-discharge profiles on the total mass loading of both electrodes (mg cm^{-2}), b) The capacities of full cell devices with high mass loadings at a current density of 1 A g^{-1} and c) the cycle performance of the $NMTiO_2$//NG full cell at a current density of 3 A g^{-1}.

S4.13. Structural stability of NMTiO2 during the charge-discharge reactions.

We carried out the analysis for the structural stability of the $NMTiO_2$ during the charge-discharge reactions through STEM measurements. The results show that the porosity and crystallinity of $NMTiO_2$ were well maintained even after the 10,000 cycles, while the pore and nanocrystal size was slightly increased.

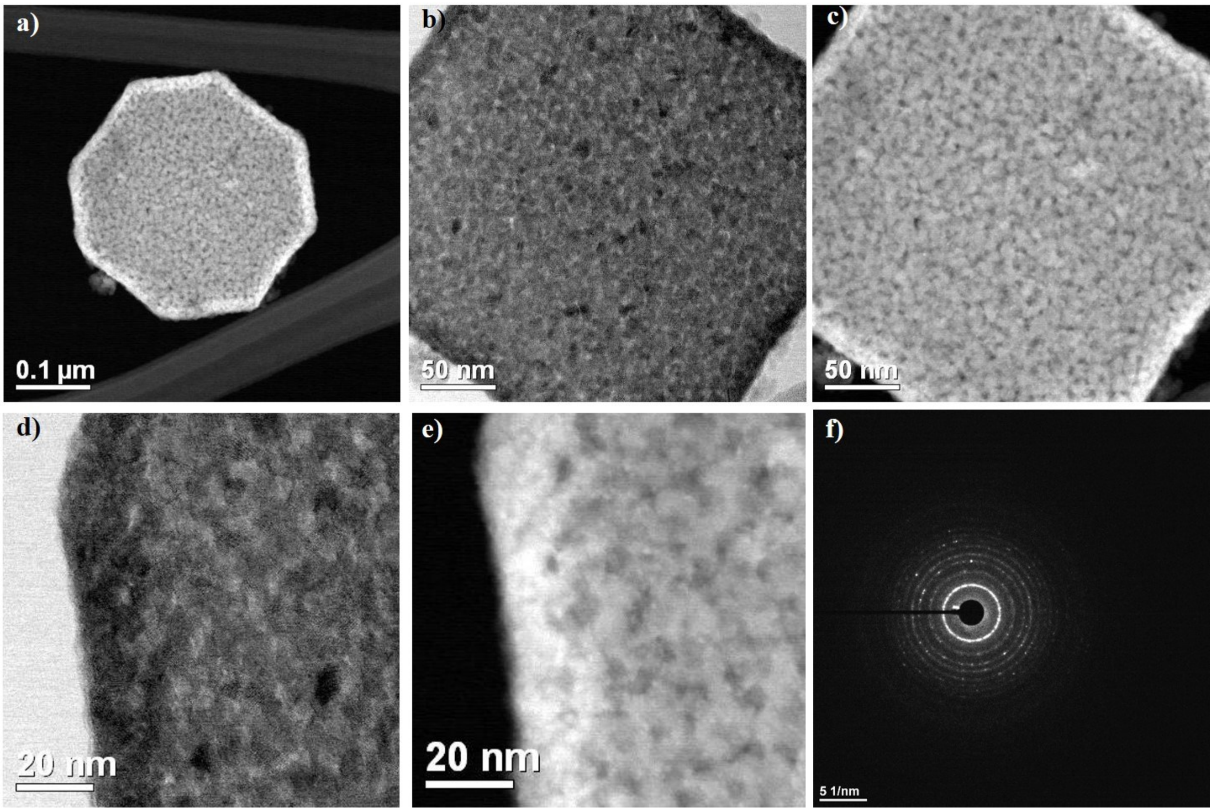

Figure S16. The porosity and crystallinity of $NMTiO_2$ after 10,000 cycles. a), b), c), d), e) The STEM images of various magnifications and f) selected area diffraction patterns of $NMTiO_2$.

S4.14. Demonstration of an ultrafast charging module

For realization of high-performance energy delivery using the $NMTiO_2$//NG energy storage full cell devices, we fabricated the USB chargeable LED module (Figure S13). These modules can be charged in the very fast charging time within 20 sec, and we find that the capacity is sufficient to operate the two blue and red led ramps.

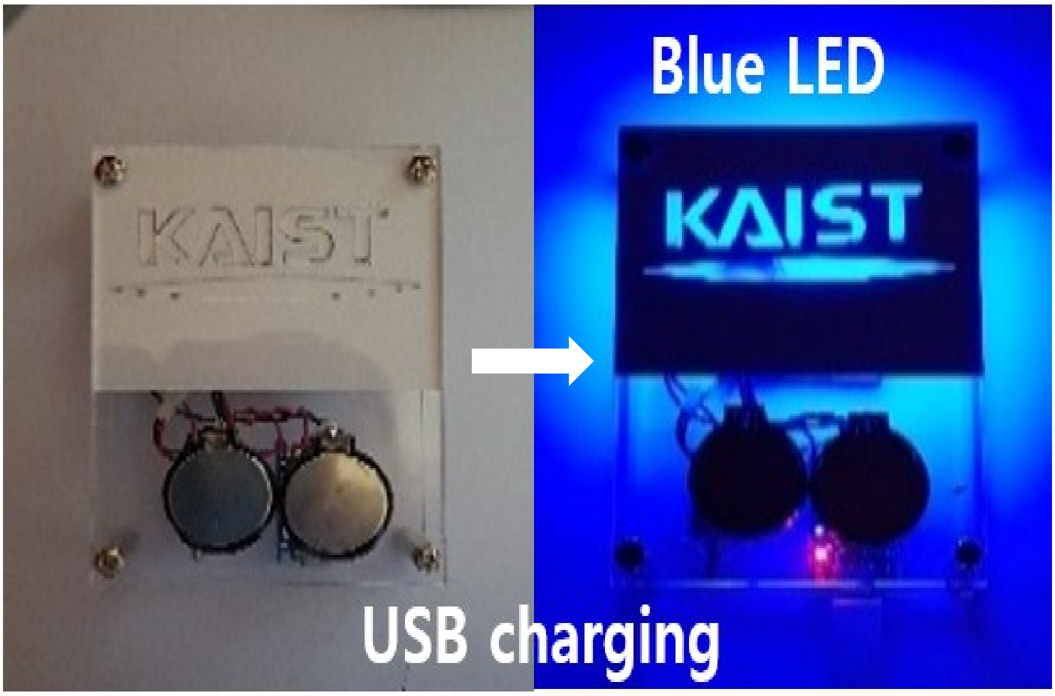

Figure S17. The USB chargeable led device demonstrating the ultrafast charging capability within 20 seconds.

	Operating potential (V)	Energy dentisy (Wh/kg)	Power density (W/kg)	Cycle
**$NMTiO_2$//NG**	1-3.8	91.1	25920	10000
**NG//NG**	0-2.5	25.6	18000	-
**$NMTiO_2$**	1-3 (VS. Na/Na^+)	-	-	10000

Table S1. The detailed information of operating conditions and performances.

References

[S1] W. S. HummersJr, R. E. Offeman, *J. Am. Chem. Soc.* **1958**, 80,1339.

[S2] G. Li, L. Li, J. Boerio-Goates and B. F. Woodfield, *J. Am. Chem. Soc.* **2005**, 127, 8659.

[S3] V. Aravindan, W. Chuiling, M. Reddy, G. S. Rao, B. Chowdari and S. Madhavi, *Phys. Chem. Chem. Phys*. **2012**, 14, 5808.

[S4] T. Sarkar, K. R. Prakasha, M. D. Bharadwaja, A. S. Prakash *Phys. Chem. Chem. Phys.* **2017**,19, 31724.
